# IgD‐Expressing Mature B Cells Exhibit Enhanced Sensitivity to Glucocorticoid‐Induced Cell Death

**DOI:** 10.1002/eji.70137

**Published:** 2026-01-25

**Authors:** Kais Almohammad, Marc Young, Sabine Vettorazzi, Franziska Greulich, Mahmoud Alkhatib, Jan Tuckermann, Hassan Jumaa, Corinna S. Setz

**Affiliations:** ^1^ Institute of Immunology Ulm University Medical Center Albert‐Einstein‐Allee 11 Ulm Germany; ^2^ Department of Cardiology University Heart Center Ulm Albert‐Einstein‐Allee 23 Ulm Germany; ^3^ Institute of Comparative Molecular Endocrinology and Physiology Ulm University Helmholtzstr. 8/1 Ulm Germany; ^4^ Chair of Metabolic Programming TUM School of Life Sciences ZIEL‐Institute For Food & Health Technische Universität München Gregor‐Mendel‐Str. 2 Freising Germany; ^5^ Department of Neurology Ulm University Medical Center Oberer Eselsberg 45 Ulm Germany; ^6^ German Center for Child and Adolescent Health (DZKJ) partner site Ulm Frauensteige 14a Ulm Germany

**Keywords:** B cells, glucocorticoids, IgD, IgM, terminal differentiation

## Abstract

Glucocorticoids (GCs) regulate diverse physiological processes, comprising metabolism, immune responses, stress adaptation, and inflammation. Synthetic GCs are widely used for their powerful anti‐inflammatory and immunosuppressive effects, in the treatment of autoimmune diseases, allergies, and inflammation. Here, we investigated the role of the glucocorticoid receptor (GR) in B cell development and survival using both B cell‐specific GR‐deficient mice and continuous *in vivo* GR agonist treatments. Deletion of the GR in B cells altered splenic B cell subpopulations, increasing follicular and CD21^lo^ B cells and leading to the accumulation of IgM^−^/IgD^−^ B cells. *In vivo* treatment with GR agonists, such as Dexamethasone (Dex) and Prednisolone (Pred), selectively depleted IgD^hi^ follicular while enriching IgM^hi^ marginal zone B cells. IgM^hi^ B cells, which were more resistant to GC‐induced cell death, showed an increased expression of IL‐10 and genes involved in survival, suggesting a potential regulatory function. *In vitro*, B cell activation via CpG or lipopolysaccharide (LPS) altered IgM/IgD expression and B cell sensitivity to GR agonists, thereby leading to improved B cell survival and increased plasma cell differentiation. Together, these findings suggest that IgD downregulation and IgM upregulation are critical for B cell survival under GC exposure and that GR agonists promote the enrichment of IgM^hi^ cells resistant to apoptosis.

## Graphical abstract

Our study shows that mature B cells expressing IgD exhibit significantly higher sensitivity to glucocorticoid (GC)‐induced cell death. This elevated sensitivity is specifically dependent on GC receptor (GR) expression and is accompanied by enhanced activation as well as terminal differentiation of B cells. Our findings elucidate how exogenous GCs affect B cell survival and suggest that their endogenous counterparts regulate B cell homeostasis and activation.

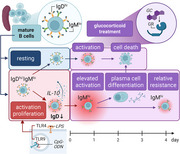

**Abbreviations**
BCR: B cell receptor
BM: bone marrow
CpG‐ODN (C): unmethylated CpG oligonucleotide(s)
ctrl: control
DCS: dead cell stain
Dex (D): Dexamethasone
dpt: days post‐treatment
Fo.B: follicular B cells
GC(s): glucocorticoid(s)
GR: glucocorticoid receptor
hi: high
IC: intracellular
LPS (L): lipopolysaccharide
lo: low
MACS: magnetic activated cell sorting
MFI: mean fluorescence intensity
MZ.B: marginal zone B cells
Pred (P): prednisolone
SAV: streptavidin
SD: standard deviation
SP: Spleen
TLR: toll‐like receptor
WT: wild‐type

## 1 Introduction

Glucocorticoids (GCs) are a class of steroid hormones that play a fundamental role in maintaining homeostasis and regulating numerous physiological processes, including metabolism, immune response, stress adaptation, and inflammation [1, 2, 3, 4, 5, 6, 7]. Produced in the adrenal cortex in response to signals from the hypothalamic–pituitary–adrenal (HPA) axis [8, 9], GCs are key mediators in adapting to environmental changes, and their broad regulatory effects encompass a variety of organs and systems, positioning them as essential players in both normal physiology and stress‐induced responses [10].

The synthesis of GCs occurs through the adrenal cortex, where cholesterol serves as a precursor molecule [8, 11, 12]. The hormone cortisol (also known as hydrocortisone) is the primary GC produced in humans, whereas corticosterone is more dominant in rodents. Upon stimulation by adrenocorticotropic hormone (ACTH) released by the pituitary gland, the adrenal glands synthesize and secrete GCs, which then circulate in the bloodstream, exerting systemic effects [13]. This tightly controlled synthesis is crucial for physiological balance; dysregulation of GC levels, either by overproduction or deficiency, can have significant effects on health.

Overproduction of GCs can lead to conditions, such as Cushing's syndrome, characterized by symptoms, including weight gain, hypertension, hyperglycemia, and immune suppression [14]. Conversely, an absence or severe deficiency of GCs, such as in Addison's disease, can result in symptoms, such as fatigue, weight loss, hypotension, and potentially life‐threatening adrenal crises [15]. Both conditions highlight the necessity of maintaining appropriate GC levels for physiological function.

To manage GC dysregulation, synthetic GCs are often administered to either supplement or replace endogenous hormone production. These synthetic compounds are highly potent, with varying degrees of efficacy compared to cortisol [16, 17]. Dexamethasone (Dex) is considered approximately 100‐fold more potent than hydrocortisone, whereas prednisolone (Pred) has about 10‐fold greater potency compared to hydrocortisone [16, 18, 19, 20]. These synthetic GCs are commonly used in clinical settings for their powerful anti‐inflammatory and immunosuppressive effects, particularly in the treatment of autoimmune diseases, allergies, hematologic neoplasms and inflammation [21, 22, 23, 24, 25, 26]. In autoimmune conditions, GCs modulate the expression of a broad range of immune response‐related genes [27]. Moreover, while inhibiting leukocyte trafficking, GCs are also thought to interfere with leukocyte activation and expansion [28] with varying effects on individual immune cell populations in different disease types [25].

GCs exert their effects primarily through binding to the glucocorticoid receptor (GR), a ligand‐activated transcription factor of the nuclear receptor superfamily [29]. Upon binding, the GC–GR complex undergoes a conformational change that allows it to translocate into the cell nucleus, where it acts as a transcription factor [30]. This complex interacts with specific glucocorticoid response elements (GREs) on DNA, leading to either activation or repression of target gene transcription [31, 32, 33]. Through this mechanism, GCs regulate the expression of numerous genes involved in immune modulation, metabolism, and cellular stress responses [34]. The GR can also interact with other transcription factors, such as NF‐κB and AP‐1, to inhibit their activity and thereby exert anti‐inflammatory effects [6, 35, 36, 37].

The immune system is a major target of GC action, and their effects on lymphoid cells, including B lymphocytes, are particularly significant. The GR is expressed in various immune cell types, comprising all developmental stages of B cells, where it mediates the direct effects of GCs on these lymphocytes [29, 38]. By producing pro‐ and anti‐inflammatory cytokines, acting as professional antigen presenting cells during T cell activation and their capacity to differentiate into (auto‐)antibody secreting and persisting memory cells, B cells function both as drivers and regulators of immune responses [39]. GCs specifically impair B cell function by reducing B cell receptor (BCR) and toll‐like receptor (TLR) signaling, decreasing transcription of immunoglobulin loci, and inducing apoptosis [38, 40, 41]. However, although GCs were shown to induce apoptosis in B and T cells [38, 41], some studies suggested that GCs enhance survival in certain contexts [42, 43, 44]. GCs have been shown to influence B cell differentiation function and survival, thereby regulating B cell homeostasis and preventing excessive immune reactions [38, 41].

Thus, despite the progress in elucidating the role of GCs and their receptor in B cell biology, several questions remain unanswered. Specifically, it remains unclear how different levels of GR activity influence B cell populations and responses under various physiological and pathological conditions. In this study, we addressed the effect of increased GC activity or absent GR expression on the survival of different B cell populations. By addressing the role of BCR expression and B cell activation, we found that IgM‐BCR expression combined with B cell activation provides relative protection against GC‐mediated cell death. Addressing these questions is crucial, as it could provide novel insights into the development of therapeutic strategies aimed at modulating B cell function in autoimmune diseases, allergies, and other immune‐related conditions.

## 2 Results

### 2.1 B Cell‐Specific GR Deletion Alters B Cell Subpopulations

To explore the role of the GR in B cell development, we generated B cell‐specific GR‐deficient mice by crossing GR floxed mice (*GR^f/f^*) with *mb1‐cre* mice, resulting in cre‐mediated gene inactivation at the pro‐B cell stage. The efficiency of GR deletion was confirmed by flow cytometry (Figure S1A,B) and PCR (Figure S1C,D), showing significantly reduced GR expression in splenic B cells from *GR^f/f^* × *mb1‐cre^+/ki^* mice compared to non‐B cells and wild‐type (WT) controls (Figure S1). We observed an increase in the frequency and absolute numbers of small CD25^+^ pre‐B cells (Figure S2A), although the deletion did not lead to an overall increase of total B cell numbers in the bone marrow (BM) (Figure S2B).

**FIGURE 1:**
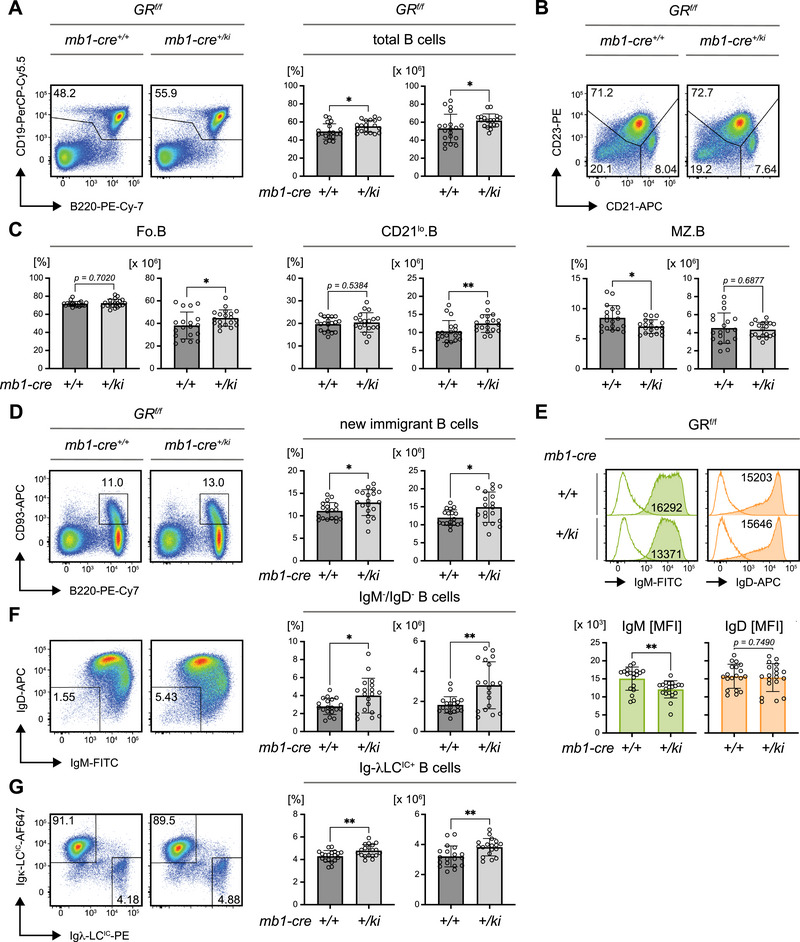
B cell–specific GR deletion alters splenic B cell subpopulations. Phenotype analyses of B cell populations in 8‐week‐old glucocorticoid receptor (GR) *GR^f/f^* × *mb1‐cre* mice of the indicated genotype. Flow cytometric data were pre‐gated on B cells as shown in Figure S1A. Unless specified otherwise, *n* = 19 per group, mean ± standard deviation (SD) and statistical significance was calculated by applying the unpaired *t* test. (A) Representative flow cytometric analysis of total splenic B cells (left panel) pre‐gated on viable single cells and quantification of percentages (bar diagrams, left) and absolute cell numbers (bar diagrams, right). (B) Representative flow cytometric analysis of follicular (Fo.B: CD23^+^/CD21^+^) marginal zone (MZ.B: CD23^−^/CD21^+^) and CD21^lo^ B (CD21^lo^.B: CD23^−^/CD21^lo^) cells in the spleens of mice of the indicated genotypes. (C) Quantified percentages and absolute cell numbers of Fo.B cells (left), CD21^lo^.B (middle) and MZ.B cells (right) in the spleens of *GR^f/^* × *mb1‐cre* mice. Statistical significance was determined using the Mann–Whitney *U*‐test to compare percentages of Fo.B and absolute cell numbers of CD21^lo^.B cells. (D) Representative flow cytometric analysis of new immigrant B cells in spleens (left panel) and quantification of percentages (bar diagrams, left) and absolute cell numbers (bar diagrams, right). *GR^f/f^* × *mb1‐cre^+/+^*, *n* = 18. (E) Representative flow cytometric analysis of IgM (green) and IgD (orange) surface expression in total splenic B cells (top panel) and quantification of the respective mean fluorescence intensities (MFI; bottom panel). Statistical significance was calculated using the Mann–Whitney *U*‐test to compare IgM MFIs. (F) Representative flow cytometric analysis of IgM^−^/IgD^−^ splenic B cells (left panel) and quantification of percentages (bar diagrams, left) and absolute cell numbers (bar diagrams, right). (G) Representative flow cytometric analysis of intracellular (IC) Ig‐κ and ‐λLC expression in splenic B cells (left panel) and quantified percentages of Ig‐λLC^+^ B cells (bar diagrams, left) and absolute cell numbers (bar diagrams, right). P values 〈 0.05 were considered as statistically significant (* p ≤ 0.05; ** p ≤ 0.01).

In the spleen, *GR^f/f^* × *mb1‐cre^+/ki^* mice displayed a higher percentage and absolute number of total B cells (Figure 1A). Further analysis of splenic B cell subpopulations revealed significant increases in the absolute numbers of follicular B cells (Fo.B) and CD21^lo^ B (CD21^lo^.B) cells, despite unchanged percentages (Figure 1B,C). Interestingly, the percentage of marginal zone B cells (MZ.B) was significantly reduced, although their absolute numbers remained unaffected (Figure 1B,C).

The CD21^lo^ B cell compartment, which includes transitional B cells and B‐1 cells, was expanded, and the population of new immigrant B cells was also elevated in GR‐deficient mice (Figure 1D). Notably, GR‐deficient B cells exhibited a reduced surface expression of IgM, whereas IgD expression was unchanged (Figure 1E). Additionally, an increased population of IgM/IgD double‐negative B cells was observed in the spleens of GR‐deficient mice (Figure 1F), accompanied by a significant accumulation of B cells expressing a lambda light chain (Ig‐λLC), whereas kappa light chain (Ig‐κLC) expressing cells remained stable (Figure 1G, Figure S2C).

Further characterization of the IgM^−^/IgD^−^ population showed that these cells were predominantly located in the CD21^lo^ B cell compartment (Figure S2D). This population likely includes plasma cells, as ELISpot assays detected IgM‐ and IgG‐secreting cells primarily within the CD21^lo^ B cell population (Figure S2E). Interestingly, the IgM^−^/IgD^−^ B cell population also contained a fraction of isotype‐switched IgG^+^ memory B cells (Figure S2F,G).

However, IgM^−^/IgD^−^ B cells from GR‐deficient mice did not show a significant increase in their ability to secrete IgM in ELISpot (Figure S2H), and the overall concentration of serum IgM and IgG remained unchanged (Figure S2I). Moreover, no significant increase in CD138^+^ plasma cells was detected in GR‐deficient mice (Figure S2J).

In conclusion, the deletion of the GR in B cells leads to changes in specific B cell subsets, including an increase in follicular and CD21^lo^ B cells, and an accumulation of IgM^−^/IgD^−^ B cells. These results suggest that the GR plays a role in regulating B cell homeostasis, possibly by eliminating certain B cell subsets that would otherwise persist in the absence of GR‐mediated signaling.

### 2.2 Continuous GC Treatment Eradicates B Cells *In Vivo*

To investigate the effects of sustained GR activation on B cells, we treated mice with the synthetic GR agonists, Dex, and Pred, using continuous‐release pellets over a period of 14 days. As Dex is approximately 10 times more potent than Pred due to differences in stability, half‐life, and bioavailability, Pred was administered at a 10‐fold higher concentration to attempt comparable effects *in vivo* [16, 18, 19, 20].

**FIGURE 2:**
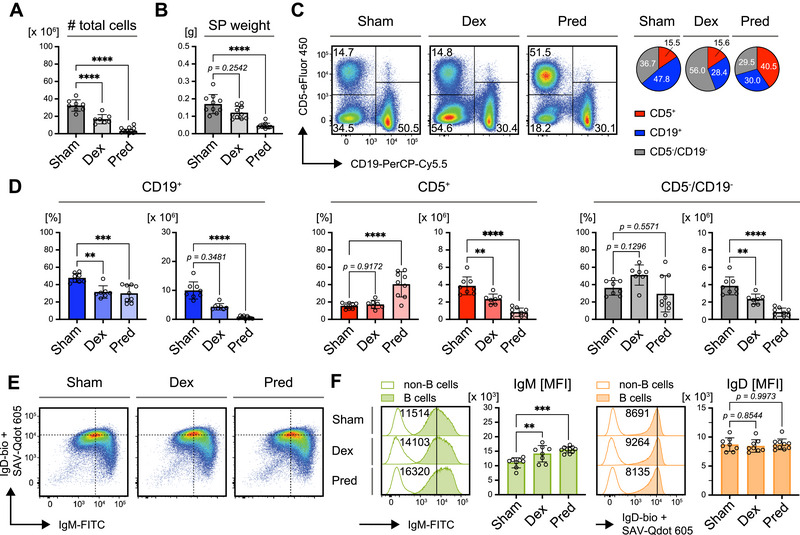
Continuous GC‐treatment eradicates B cells *in vivo*. Phenotype analyses of mice transplanted with constant glucocorticoid (GC)‐release pellets for 14 days. (A) Total cell numbers in spleens from mice following 14 days of exposure to Dexamethasone (Dex, *n* = 7), Prednisolone (Pred, *n* = 8) or control (Sham, *n* = 10) pellets. Mean ± SD. Statistical significance was calculated by applying the ordinary one‐way ANOVA. (B) Quantified spleen weights of mice after 14 days of exposure to Dex (*n* = 10), Pred (*n* = 12), or Sham (*n* = 10) pellets. Mean ± SD. Statistical significance was calculated by using the Kruskal–Wallis test. (C) Representative composition of residual cells in spleens after 14 days of treatment with the indicated pellets as measured by flow cytometry (left panel). Cells were pre‐gated on viable single lymphocytes. Pie charts in the right panel show average percentages of B cells (CD19^+^), T cells (CD5^+^), and non‐B/T cells (CD5^−^/CD19^−^), representative of ≥7 individual mice per group. (D) Quantification of percentages (left panels) and absolute cell numbers (right panels) of B cells (CD19^+^, blue), T cells (CD5^+^, red) and non‐B/T cells (CD5^−^/CD19^−^, gray) in murine spleens after 14 days of treatment with Sham (*n* = 8), Dex (*n* = 7) or Pred (*n* = 9) pellets. Mean ± SD. Statistical significance was calculated using the ordinary one‐way ANOVA for T and non‐B/T cells and the Kruskal–Wallis test for B cells, respectively. (E) Representative flow cytometric analysis of IgM and IgD surface expression in total splenic B cells (pre‐gated on viable single lymphocytes) purified from mice of the indicated treatment cohorts. (F) Representative comparison of IgM (green, left) and IgD (orange, right) MFI in total splenic B cells (pre‐gated on viable single lymphocytes) from mice of the indicated treatment groups (histograms). Non‐B cells within the respective samples served as negative controls. Bar diagrams show quantification of IgM and IgD MFI in Sham‐ (*n* = 8), Dex‐ (*n* = 7) and Pred‐treated (*n* = 9) mice. Mean ± SD. Statistical significance was calculated by using the ordinary one‐way ANOVA. P values 〈 0.05 were considered as statistically significant (** p ≤ 0.01; *** p ≤ 0.001; **** p ≤ 0.0001).

Both Dex and Pred significantly reduced the percentage of total B cells in the BM (Figure S3A) and total splenocyte numbers compared to Sham‐treated controls (Figure 2A). However, Pred treatment was more effective, as shown by a significant reduction in spleen weight (Figure 2B) and absolute B cell numbers in the BM, which was not observed upon Dex treatment (Figure S3A). Additionally, both treatments led to a significant suppression of endogenous corticosterone levels, suggesting interference with the body's natural GC biosynthesis (manuscript in preparation).

To assess the remaining lymphocyte populations after 14 days of treatment, splenocytes were stained with anti‐CD5 and ‐CD19 antibodies (Figure 2C). The frequency of B cells, which typically represent about half of the splenic cells in sham‐treated controls, was drastically reduced to less than one‐third of the remaining cells. Interestingly, although the total numbers of T cells were reduced, their frequency in the residual splenic cell population remained constant or was even increased following Pred treatment (Figure 2D). Non‐B/non‐T cells also showed a trend toward an increased frequency in Dex‐treated mice, indicating that T cells and non‐B/non‐T cells were more resistant to GC‐induced depletion than B cells and that T cells also tolerate a high concentration of Pred.

Given the previously observed reduction in IgM expression following GR inactivation (Figure 1E), we analyzed IgM and IgD expression on B cells that survived GC treatment. The IgM/IgD profile resembled that of Sham‐treated controls (Figure 2E), but a notable shift towards higher IgM expression was observed in GR agonist‐treated mice. Flow cytometric analysis revealed that IgM expression was significantly upregulated following treatment with GR agonists (Figure 2F), contrasting with the decreased IgM levels seen in GR‐deficient B cells. Although IgD expression remained unchanged, the increase in surface IgM significantly raised the IgM/IgD ratio (Figure S3B).

In conclusion, these results indicate that continuous GC exposure selectively favors the survival of IgM^hi^ B cells, potentially as a mechanism to escape GC‐mediated cell death. Additionally, B cells appear to be highly sensitive to GC‐induced depletion compared to other immune cells, such as T cells and non‐lymphoid/myeloid cells, which are less affected under the same treatment conditions.

### 2.3 Activated B Cells Display Relative Resistance to Treatment With GR Agonists

**FIGURE 3:**
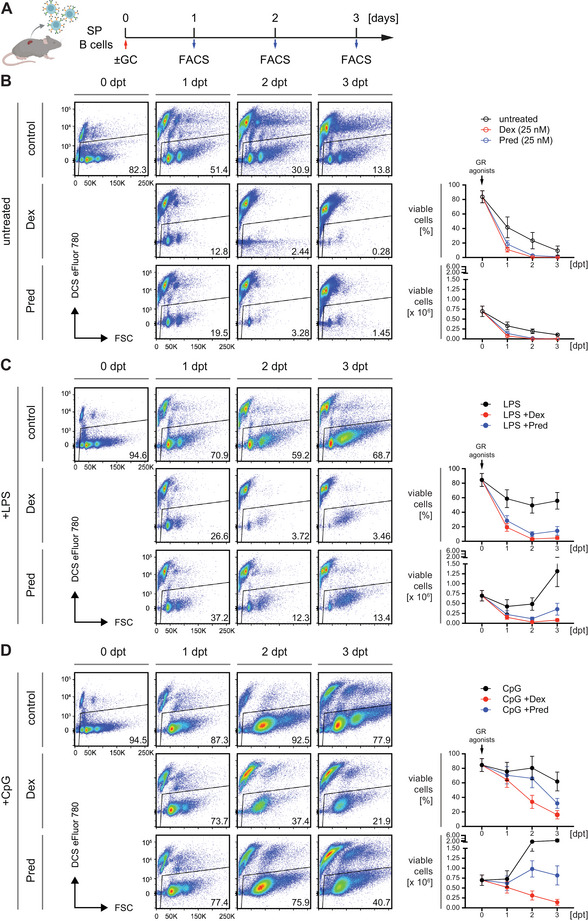
Activated mature B cells are more resistant to GR agonist treatment *in vitro*. (A) Schematic timeline of the experimental procedure: Mature splenic B cells from wild‐type (WT) mice were purified by negative selection using magnetic activated cell sorting (MACS) and treated with the indicated GR agonists at a concentration of 25 nM. Cell viability was monitored by flow cytometry from days 1 to 4 post treatment (dpt). (B) Representative flow cytometric analyses of cell viability following treatment with GR agonists (left panel) performed *in vitro* as described in (A). Quantified percentages (right panel, top) and absolute cell numbers (right panel, bottom) of viable cells. Numbers of replicates are listed in Table S3, mean ± SD. (C) Representative flow cytometric analyses of cell viability following simultaneous treatment with 2.5 µg/mL lipopolysaccharide (LPS) and GR agonists (left panel), performed *in vitro* as described in (A). Quantified percentages (right panel, top) and absolute cell numbers (right panel, bottom) of viable cells. Numbers of replicates are listed in Table S4, mean ± SD. (D) Representative flow cytometric analyses of cell viability following simultaneous treatment with 2 µM CpG‐ODN #1826 (CpG) and GR agonists (left panel), performed *in vitro* as described in (A). Quantified percentages (right panel, top) and absolute cell numbers (right panel, bottom) of viable cells. Numbers of replicates are listed in Table S5, mean ± SD.

To further investigate the effects of GR activation on B cells, we purified mature splenic B cells from WT mice and treated them *in vitro* with the GR agonists Dex or Pred (Figure 3A). Even at a low nanomolar concentration (25 nM), GR agonist exposure led to the complete eradication of all viable B cells within 3 days (Figure 3B). In contrast, mature B cells from *GR^f/^* × *mb1‐cre^+/ki^* mice, which lack functional GRs in B cells, were resistant to the treatment (Figure S4A), confirming that the cell loss was specifically mediated by GR activation.

To address the effect of cell activation on GC‐mediated cell death, we used the TLR4 agonist lipopolysaccharide (LPS) to simultaneously activate B cells during GR agonist treatment (Figure 3C, Figure S4B). LPS activation of B cells led to a minor improvement in survival compared to resting cells, with a small proportion surviving until day 3 of treatment (Figure S4C,E). As TLR9 activation by unmethylated CpG oligonucleotides (CpG) typically induces stronger and faster activation than LPS, we also tested whether CpG could better protect B cells from GR agonist‐mediated cell death (Figure 3D). Remarkably, co‐stimulation with CpG significantly rescued B cells from death when applied simultaneously with GCs, further demonstrating that activated B cells are more resistant to GR‐induced cell death than resting B cells (Figure S4C,D,E).

These findings suggest that although both LPS and CpG act through MyD88‐dependent pathways [45], the activation profiles they induce may differ in strength, speed, and their capacity to rescue B cells from GC‐induced apoptosis. Moreover, resting B cells isolated from the spleen and cultured *in vitro* in the presence of GR agonists are highly sensitive to GC‐mediated cell death, but activation via TLRs significantly improves survival.

### 2.4 GR Agonists Enhance B Cell Activation and Accelerate Terminal Differentiation

The data above allow the conclusion that CpG provides more effective protection through rapidly and robustly activating B cells, thereby enabling them to withstand GC‐induced apoptosis. To confirm this conclusion, we analyzed the expression of the activation markers CD69 and CD86 on WT B cells treated with either TLR ligand (Figure S5A). CpG treatment led to rapid and nearly complete upregulation of CD69, with >90% of cells showing elevated expression 1 day post‐treatment (dpt). In contrast, LPS treatment resulted in elevated CD69 expression in <50% of cells. Interestingly, although CD86 expression remained low and declined in CpG‐treated cells over time, LPS‐treated B cells exhibited a marked upregulation of CD86, which increased steadily until 3 dpt (Figure S5A).

**FIGURE 4:**
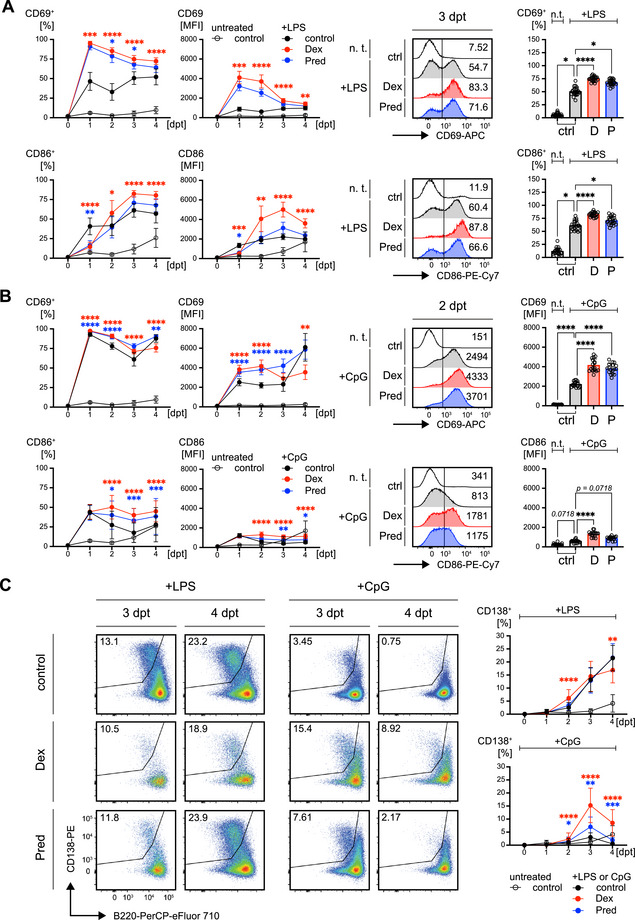
GR agonists enhance B cell activation and accelerate terminal differentiation. (A and B) Kinetics of the activation markers CD69 (top panel) and CD86 (bottom panel), determined by flow cytometry in WT splenic B cells following treatment with GR agonists as described in Figure 3A in the presence of LPS (A) or CpG (B). Representative flow cytometric analyses and bar diagrams on the right‐hand side compare percentages of CD69 and CD86 positive cells upon LPS‐treatment at 3 dpt (A) or the respective MFIs for CpG‐treatment at 2 dpt (B, MFIs shown since, in contrast to LPS, all CpG treated cells showed immediate upregulation of CD69). (A) Numbers of replicates are listed in Tables S6 and S7, mean ± SD. Statistical significance was calculated by applying either the repeated measures (RM) one‐way ANOVA or the Friedman test, respectively. Asterisks in the kinetics indicate significant differences between cells stimulated with LPS or CpG alone and cells additionally treated with GR agonists Dex (D, red asterisks) or Pred (P, blue asterisks). (C) Representative flow cytometric analysis of *in vitro* plasma cell differentiation at days 3 and 4 following treatment of WT splenic B cells with GCs in the presence of either LPS (left) or CpG (right). The changes in CD138^+^/B220^lo^ cells over time are shown as kinetics on the right‐hand side. For all groups and time points, *n* = 23, except for 3 dpt (*n* = 22), mean ± SD. Statistical significance was calculated by applying either the RM one‐way ANOVA or the Friedman test, respectively. Asterisks in the kinetics indicate significant differences between cells stimulated with LPS or CpG alone and cells additionally treated with GR agonists Dex (D, red asterisks) or Pred (P, blue asterisks).

When combined with GR agonist treatment (Dex or Pred), both LPS‐ and CpG‐activated B cells showed a significant enhancement in the expression of CD69 and CD86 (Figure 4A,B). Interestingly, treatment of splenic B cells with GR agonists in the absence of LPS or CpG, already resulted in an upregulation of the activation marker CD69 at 1 and 2 dpt, whereas CD86 was only upregulated at day 2 in the few residual cells (Figure S5B). Following concomitant treatment with LPS, CD69 expression was significantly increased upon addition of Dex or Pred, whereas CD86 was decreased following Dex or Pred treatment on day 1 and later increased on 3 dpt (Figure 4A). On the other hand, CpG‐treated B cells exhibited an increased expression of both CD69 and CD86, which was not further increased at 1 day after Dex or Pred treatment (Figure 4B). However, on the following days, Dex or Pred treatment led to an increase in CD86 expression as compared with cells treated only with CpG. Together, these data suggest that rapid and sustained CD69 upregulation distinguishes CpG‐ from LPS‐induced splenic B cells and that GC treatment selects these cells.

Next, we explored whether the addition of GR agonists influences plasma cell differentiation, which is typically induced by LPS *in vitro*. Although Dex or Pred treatment did not further enhance the plasma cell differentiation capacity of LPS‐stimulated B cells (Figure 4C), an interesting observation was made in CpG‐treated cells: Although CpG alone was insufficient to induce efficient terminal differentiation, the addition of GR agonists led to the generation of CD138^+^/B220^lo^ plasma cells (Figure 4C).

Together, the data suggest that CpG and LPS differ in their kinetics of B cell activation and that GR agonists amplify this activation. Moreover, the GR agonists boosted plasma cell differentiation of B cells treated with CpG, which was not observed for LPS.

### 2.5 Delayed GR Agonist Treatment After LPS Activation Improves B Cell Survival

The data presented above suggested that B cell activation plays a crucial role in protecting cells from GC‐induced cell death. On the basis of CD69 expression, CpG was shown to induce faster B cell activation than LPS, likely explaining its superior ability to protect B cells during simultaneous treatment with GR agonists. Therefore, we hypothesized that LPS might offer protection if GR agonist treatment is delayed, allowing sufficient time for activation to occur.

**FIGURE 5:**
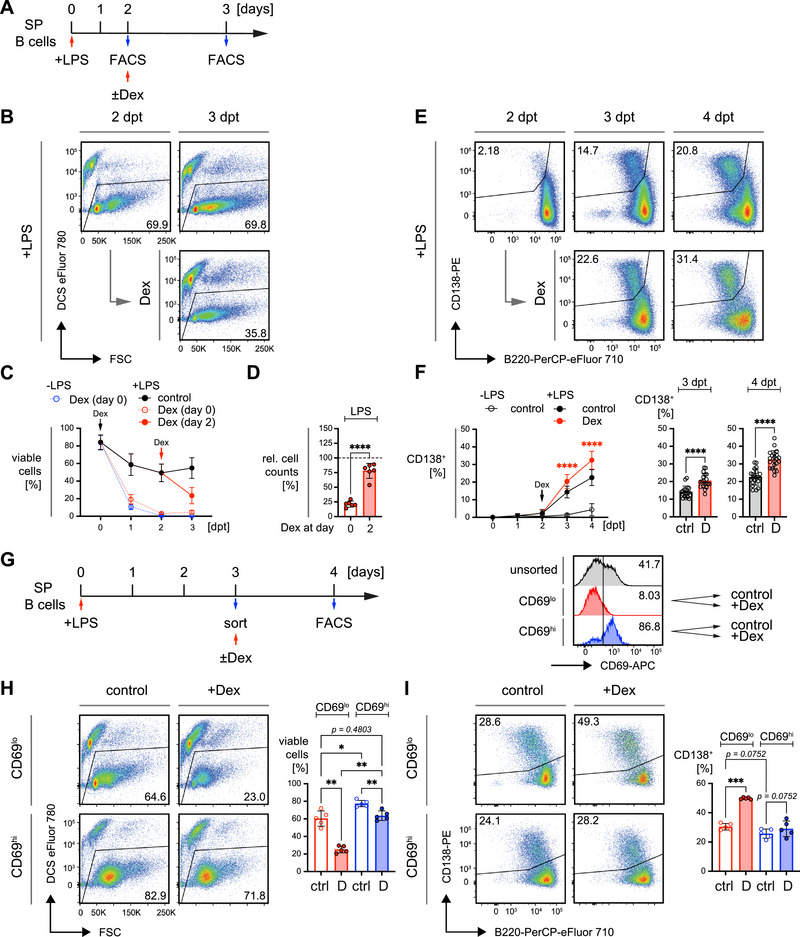
Delayed GR agonist treatment after LPS activation improves B cell survival. (A) Schematic timeline of the experimental procedure: Mature splenic B cells from WT mice were isolated by negative selection via MACS and treated with LPS. On day 2, Dex (D) was added, or the cells remained untreated (ctrl). Cell viability was monitored by flow cytometry 1 day later (3 dpt). (B) Representative flow cytometric analyses of cell viability at days 2 and 3 in LPS pre‐stimulated B cells upon addition of Dex at day 2. (C) Kinetics comparing survival of mature B cells when treated with Dex at day 0 (dotted lines, data already shown in Figure 3B) in the presence (red) or absence of LPS (blue) to survival, when Dex was added at day 2 (solid lines and filled symbols). Numbers of replicates are listed in Table S9, mean ± SD. (D) Relative change of absolute cell numbers following 1 day of Dex treatment. Cells were treated overnight either with LPS and Dex at day 0, or after 2 days of LPS‐pre‐treatment (Dex at day 2). Cell counts were normalized to the respective cell count before overnight Dex‐treatment. *n* = 6 per group, mean ± SD. Statistical significance was determined using the paired *t* test. (E) Representative flow cytometric analyses of plasma cell differentiation at the indicated time points in LPS pre‐stimulated B cells upon delayed addition of 25 nM Dex, as described in (A). (F) Kinetics (left panel) show quantified percentages of CD138^+^/B220^lo^ cells when Dex (D) was added on day 2 upon pre‐stimulation with LPS. Bar diagrams (right panel) compare percentages of CD138^+^/B220^lo^ cells in the indicated treatment groups at 3 and 4 dpt. Numbers of replicates are listed in Table S10, mean ± SD. Statistical significance was calculated by applying the Friedman test or RM one‐way ANOVA for assessing differences at days 3 and 4, respectively. Asterisks in the kinetics indicate significant differences between cells stimulated with LPS alone and cells additionally treated with GR agonists Dex (D, red asterisks). (G) Schematic timeline of the experimental procedure: Mature splenic B cells from WT mice were purified by negative selection via MACS and treated with LPS. On day 3, CD69^lo^ and CD69^hi^ B cells were FACS‐purified according to the gating strategy shown in Figure S7A. Histograms on the right depict representative re‐analysis of CD69 expression, indicating the percentages of CD69^+^ cells prior to and after sorting. Purified cells were treated overnight in the presence (D) or absence (ctrl) of Dex, *n* = 5, mean ± SD. Cell viability was monitored by flow cytometry 1 day later (4 dpt). (H) Representative flow cytometric analyses of cell viability in FACS‐purified CD69^lo^ and CD69^hi^ B cells upon treatment with Dex (D, left panel) as described in (G) and quantified percentages of viable cells, *n* = 5 in each group, mean ± SD. Statistical significance was calculated by applying the RM one‐way ANOVA. (I) Representative flow cytometric analyses of *in vitro* plasma cell differentiation in FACS‐purified CD69^lo^ and CD69^hi^ B cells upon treatment with Dex (D, left panel) described in (G) and quantified percentages of CD138^+^/B220^lo^ cells, *n* = 5 in each group, mean ± SD. Statistical significance was calculated by applying the RM one‐way ANOVA.

To directly test this, splenic B cells were pre‐incubated with LPS, and GR agonists were added 2 days later (Figure 5A). The delayed GR agonist treatment resulted in substantial rescue of the LPS‐activated B cells at 3 dpt (Figure 5B–D, Figure S6A,B), compared to simultaneous treatment with LPS and GR agonists. Moreover, a delayed treatment of LPS‐stimulated cells with GR agonists led to a significant increase in the proportion of plasma cells observed on days 3 and 4 (Figure 5E,F). Notably, GR‐deficient splenic B cells were also resistant to delayed treatment with either Dex or Pred (Figure S6C) and did not show the enhanced plasma cell differentiation as compared with cells from control mice (Figure S6D).

To determine whether LPS‐induced activation was responsible for the improved survival of B cells, we sorted LPS‐activated B cells on the basis of CD69 expression on day 3 (Figure S7A; Figure 5G). Time‐course experiments had previously revealed that CD69 expression peaked on this day (Figure S5A). Subsequent addition of Dex to those sorted cells further increased CD69 expression in CD69^lo^ and CD69^hi^ populations, whereas CD86 levels remained unchanged (Figure S7B). Moreover, GR agonist treatment demonstrated that CD69^hi^ B cells were significantly more resistant to GC‐mediated cell death compared to CD69^lo^ cells (Figure 5H). Remarkably, Dex‐treated CD69^hi^ cells exhibited survival rates similar to untreated CD69^lo^ cells, suggesting that activation confers CD69^hi^ cells a relative resistance to GC‐induced cell death.

Interestingly, when examining plasma cell differentiation, GR agonist treatment induced a significant increase in plasma cells in the CD69^lo^ population but not in the CD69^hi^ population (Figure 5I). Together, these data suggest that the induction of activation markers, such as CD69, is associated with protection from GC‐mediated cell death, whereas cells lacking activation markers show an increased propensity for plasma cell differentiation upon GR agonist treatment.

### 2.6 IgD‐BCR Expression Affects Resistance to Cell Death

Similar to the regulation of activation markers like CD69 and CD86, CpG treatment led to distinct kinetics in the modulation of IgM‐/IgD‐BCR expression on splenic B cells *ex vivo*. Specifically, CpG induced a rapid downregulation of IgD in the entire cell population, whereas LPS caused a delayed yet sustained downregulation of IgD only in a subfraction of the *in vitro*/*ex vivo* cultured splenic B cells (Figure S7C). This difference in BCR expression mirrored the kinetics observed with activation markers and seemed to impact how B cells responded to GR agonist treatment (Figure S5A).

**FIGURE 6:**
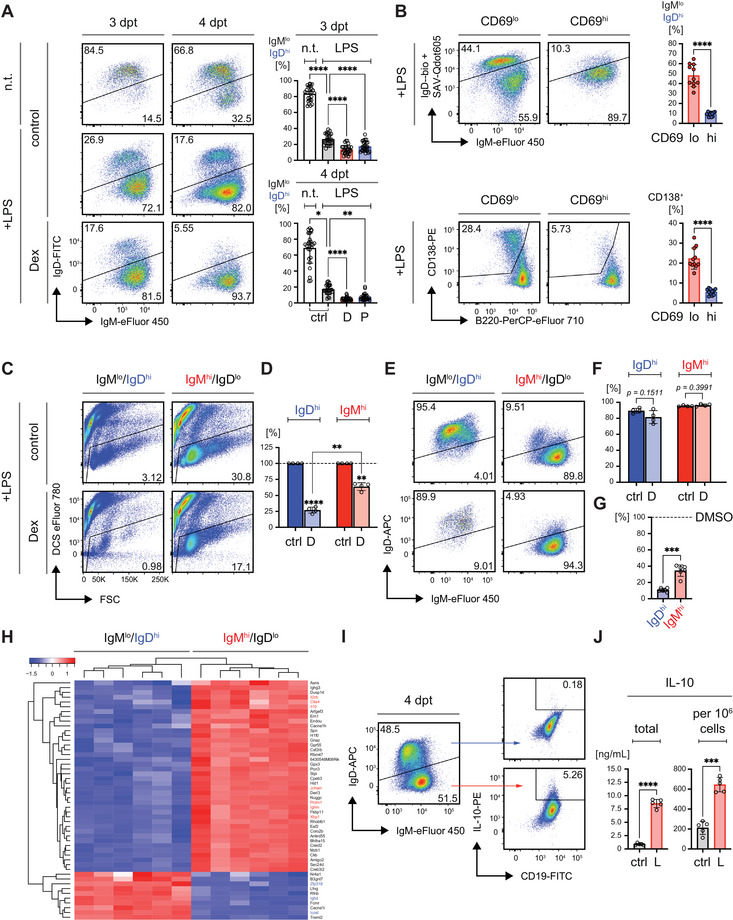
IgD‐BCR expression affects resistance to cell death. (A) Representative flow cytometric analyses of IgM and IgD surface expression in mature splenic B cells upon stimulation with LPS and delayed treatment with GR agonists as described in Figure 5A. Bar diagrams below depict quantified percentages of B cells with IgM^lo^/IgD^hi^ surface expression at 3 and 4 dpt. For day 3: *n* = 22; day 4: *n* = 27; mean ± SD. Statistical significance was calculated by applying the RM one‐way ANOVA or the Friedman test, respectively. (B) Representative flow cytometric analyses of IgM/IgD surface expression (top) and plasma cell differentiation (CD138/B220 bottom) in CD69^hi^ and CD69^lo^ splenic WT B cells at day 3 upon stimulation with LPS. Bar diagrams next to the flow cytometry data show quantified percentages of IgD^hi^ cells and CD138^+^ cells in the indicated cell populations; *n* = 11, mean ± SD. Statistical significance was calculated by applying the paired *t* test. (C) Representative flow cytometric analysis of viability in FACS‐purified IgM^lo^/IgD^hi^ and IgM^hi^/IgD^lo^ mature splenic B cells at 3 dpt upon sorting and treatment with Dex as indicated in the schematic timeline displayed in Figure 5G. Mature splenic B cells from WT mice were purified by negative selection via MACS and stimulated with LPS. After 2 days of incubation, cells were FACS‐purified to separate IgM^lo^/IgD^hi^ and IgM^hi^/IgD^lo^ populations according to the gating strategy in Figure S8A. Subsequently, Dex was added to the culture and viability and BCR expression were monitored by flow cytometry at 3 dpt. (D) Relative percentages of viable cells following LPS and Dex‐treatment from (C) normalized to viability in LPS‐treated cells, both determined at 3 dpt, *n* = 4, mean ± SD. Statistical significance between control‐ and Dex (D)‐treated cells was calculated by applying the one‐sample *t* test, whereas differences between D‐treated IgM^lo^/IgD^hi^ and IgM^hi^/IgD^lo^ were assessed by using the RM one‐way ANOVA. (E) Representative flow cytometric analysis of IgM and IgD surface expression in purified mature splenic B cells at 3 dpt upon sorting and Dex treatment as described in (D). (F) Quantified percentages of IgM^lo^/IgD^hi^ (blue) and IgM^hi^/IgD^lo^ (red) in purified B cells from (E). *n* = 4, mean ± SD. Statistical significance between control‐ and Dex (D)‐treated cells was calculated by applying the RM one‐way ANOVA. (G) Relative change of absolute cell numbers in IgM^lo^/IgD^hi^ (blue) and IgM^hi^/IgD^lo^ (red) cells normalized to the respective cell counts in DMSO‐treated samples in cells from the experiment shown in (A). *n* = 5 per group, mean ± SD. Statistical significance was determined using the paired *t* test. (H) Heatmap of the 50 most up‐ (red) or downregulated (blue) transcripts in IgM^lo^/IgD^hi^ and IgM^hi^/IgD^lo^ splenic B cells from WT mice as determined by RNA deep sequencing. Mature splenic B cells from WT mice (*n* = 6) were purified by negative selection via MACS and stimulated with LPS. After 3 days of incubation, cells were FACS‐purified to separate IgM^lo^/IgD^hi^ and IgM^hi^/IgD^lo^ populations for transcriptome analysis, data also listed in Table S11. (I) Representative flow cytometric analysis of IL‐10 expression at 4 dpt in IgM^lo^/IgD^hi^ and IgM^hi^/IgD^lo^ splenic WT B cells upon cultivation in the presence of LPS. (J) Total (left) and cell number‐adjusted (right) concentrations of IL‐10 determined by cytometric bead array (CBA) in cell culture supernatants from purified mature splenic B cells left untreated or cultured in the presence of LPS (L) for 4 days, *n* = 5. Statistical significance was determined by using the paired *t*‐test. P values 〈 0.05 were considered as statistically significant (* p ≤ 0.05; ** p ≤ 0.01; *** p ≤ 0.001; **** p ≤ 0.0001).

When we treated the LPS‐stimulated cells with GR agonists, IgD^hi^ B cells proliferated less efficiently and were particularly susceptible to GC‐induced cell death (Figure 6A) in a GR‐dependent manner (Figure S7D). In contrast, IgM^hi^ (IgD^lo^) cells, which increased following LPS treatment, were further enriched when exposed to GR agonists. This pattern suggested that IgD downregulation, particularly in IgM^hi^ B cells, was linked to improved survival under LPS treatment (Figure 5D).

Importantly, IgM^hi^ (IgD^lo^) expression correlated with higher CD69 expression and decreased plasma cell differentiation (Figure 6B), suggesting that the enhanced survival of CD69^hi^ B cells might be partially caused by reduced IgD‐BCR expression. In fact, IgD^hi^ cells showed increased cell death on days 3 and 4 after Dex treatment (Figure 6A).

To test whether IgD downregulation was critical for protection from GC‐induced cell death, we examined the survival of sorted IgD^hi^ (IgM^lo^) and IgM^hi^ (IgD^lo^) splenic B cells on day 2 after LPS treatment *in vitro* (Figure S8A). These sorted populations were then subjected to overnight Dex treatment. The results revealed significantly higher levels of cell death in IgD^hi^ (IgM^lo^) cells compared to IgM^hi^ (IgD^lo^) cells, suggesting that the IgM/IgD‐BCR expression ratio plays a crucial role in determining susceptibility to GC‐induced cell death (Figure 6C,D).

To investigate whether Dex directly influences IgM‐/IgD‐BCR expression or merely induces the enrichment of the IgM^hi^ (IgD^lo^) population, we analyzed BCR expression after overnight treatment with Dex. The data showed that cells expressing IgD did not downregulate IgD‐BCR but were selectively eliminated by the treatment, resulting in an increased proportion of IgM^hi^ (IgD^lo^) cells (Figure 6E–G).

These findings suggest that IgD downregulation in activated B cells is crucial for their protection from GC‐induced cell death, highlighting the importance of the IgM^hi^/IgD^lo^ phenotype in conferring resistance to GC‐induced apoptosis.

### 2.7 IgD^lo^ B Cells Show Enhanced Survival and Increased IL‐10 Expression

To further understand the differences between IgD^hi^ (IgM^lo^) and IgM^hi^ (IgD^lo^) cells, we sorted *in vitro* LPS‐activated splenic B cells on the basis of their IgM/IgD expression (Figure S8A) and performed transcriptome analysis through deep sequencing. Comparing the respective subpopulations purified from 6 individual mice, we found that the expression of genes associated with plasma cell differentiation, such as *Prdm1*, *J chain*, and *Xbp1*, was significantly upregulated in IgM^hi^ (IgD^lo^) cells (Figure 6H, Table S11 positions 8, 2, and 44, respectively). In contrast, genes necessary for IgD expression, including *Zfp318* and *Ighd*, were upregulated in IgD^hi^ (IgM^lo^) cells (Figure 6H, Table S11 positions 10 and 3, respectively).

To investigate the mechanism underlying the improved survival of IgM^hi^ (IgD^lo^) B cells, we analyzed their altered gene expression compared with IgD^hi^ (IgM^lo^) cells. Although there were no clear differences in pro‐ or anti‐apoptotic genes at the transcriptional level, we observed upregulated the expression of genes associated with T cell survival and proliferation, such as *Il2rb* and *CD28*, in the IgM^hi^ (IgD^lo^) population (Figure 6H, Table S11 positions 27 and 52, respectively). In line with these findings, KEGG pathway analysis revealed distinct differences in cytokine signaling pathways between the two cell populations (Figure S8B–D).

To validate these observations, we performed FACS staining with specific antibodies. Interestingly, IgM^hi^ (IgD^lo^) cells also exhibited increased expression of IL‐10, a cytokine known for its role in regulating immune responses (Figure 6H, Table S11 position 7). This finding is particularly notable in light of previous research showing that GCs promote the survival of anti‐inflammatory macrophages involved in resolving inflammation [42]. Together, our data suggest that GR agonists might selectively enrich for anti‐inflammatory or regulatory B cells producing IL‐10 or induce IL‐10 expression directly.

To validate this, we performed intracellular staining for IL‐10 on LPS‐activated cells and found that IgM^hi^ (IgD^lo^) cells contained and secreted higher amounts of IL‐10 (Figure 6I,J). Similar to IL‐10, the expression of CD122 (encoded by *Il2rb*) was also elevated on protein level in IgM^hi^ (IgD^lo^) B cells as compared with IgD^hi^ (IgM^lo^) following LPS stimulation (Figure S9A). Together with the IL‐2 receptor alpha chain (CD25) and the common gamma chain (CD132), CD122 (IL‐2 receptor beta chain) forms the IL‐2 receptor complex [46]. However, neither supplementation with IL‐2 nor IL‐10 showed significant effects on cell survival in the presence of GCs (Figure S9B,C).

These results indicate that IgD downregulation is crucial for protecting B cells from GC‐induced cell death by activation of cytokine signaling and that GR agonist treatment promotes the enrichment of IgM^hi^ (IgD^lo^) cells, which may possess regulatory B cell functions on the basis of their IL‐10 expression.

### 2.8 Selective Effects of GR Agonist Treatment on IgD‐Expressing B Cells *In Vivo*

The previous results suggested that B cells with increased IgD expression are more sensitive to GC‐induced cell death, whereas those with downregulated IgD‐BCR expression are more resistant. To confirm this finding *in vivo*, we analyzed splenic B cells from mice treated with Dex or Pred release pellets shown above.

**FIGURE 7:**
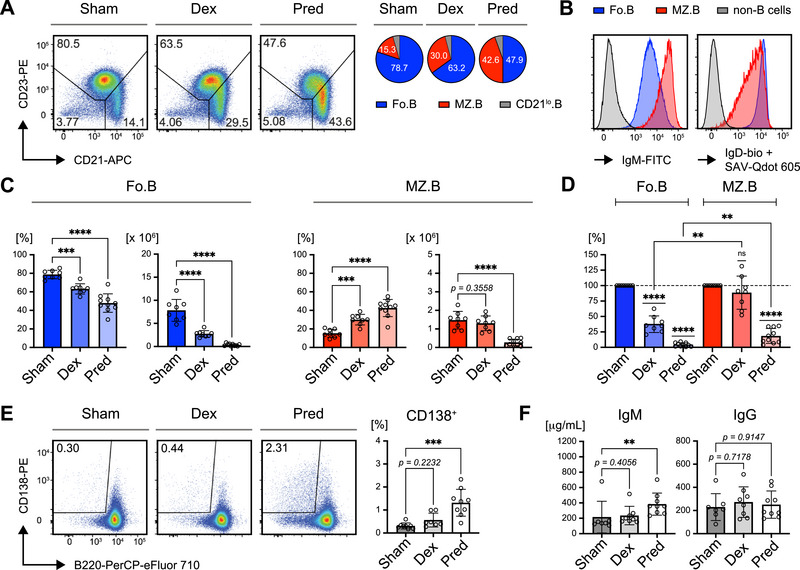
Selective effects of GR agonist treatment on IgD‐expressing B cells *in vivo*. Phenotype analyses of mice transplanted with constant GC‐release pellets for 14 days. (A) Representative flow cytometric analysis of IgM and IgD surface expression in the B cell subpopulations Fo.B, MZ.B and CD21^lo^.B, following GC treatment *in vivo*. Pie charts in the right panel show average percentages of the respective B cell subpopulations, representative of at least eight individual mice per group. (B) Representative flow cytometric analysis of the splenic B cell compartments Fo.B (CD23^+^/CD21^+^), MZ.B (CD23^−^/CD21^+^), and CD21^lo^.B (CD23^−^/CD21^lo^) after 14 days of treatment with the indicated pellets (left panel). Pie charts in the right panel show average percentages of Fo.B, MZ.B and CD21^lo^.B cells, representative of at least eight individual mice. (C) Quantification of both percentages (left) and absolute cell numbers (right) of Fo.B cells (blue) and MZ.B cells (red) in murine spleens after 14 days of treatment with Sham (*n* = 8), Dex (*n* = 8) or Pred (*n* = 10) pellets. Mean ± SD. Statistical significance was calculated by using the ordinary one‐way ANOVA or the Kruskal–Wallis test, respectively. (D) Absolute cell numbers of Fo.B cells (blue) and MZ.B cells (red) in murine spleens after 14 days of treatment with Dex (*n* = 8) or Pred (*n* = 10) pellets as shown in (C), normalized to the respective cell numbers in spleens from Sham‐treated mice (*n* = 8). Mean ± SD. Statistical significance was calculated by applying the one‐sample *t* test or a mixed‐effects analysis, respectively. (E) Representative flow cytometric analysis of plasma cells in spleens from mice of the indicated treatment groups (left panel), pre‐gated on single viable CD19^+^ cells, and quantification of percentages. Sham: *n* = 8; Dex: *n* = 7; Pred: *n* = 9; mean ± SD. Statistical significance was calculated by applying the Kruskal–Wallis test, respectively. (F) Serum IgM and IgG concentrations in mice of the indicated treatment groups determined by ELISA. Sham: *n* = 7; Dex: *n* = 8; Pred: *n* = 9; mean ± SD. Statistical significance was calculated by applying the ordinary one‐way ANOVA or the Kruskal–Wallis test, respectively. P values 〈 0.05 were considered as statistically significant (** p ≤ 0.01; *** p ≤ 0.001; **** p ≤ 0.0001).

After 14 days of GR agonist treatment, we observed a selective reduction in follicular B cells (Fo.B), which was proportional to the potency of the GR agonist used (Figure 7A). Follicular B cells, known for their higher IgD expression, were particularly sensitive to GR‐mediated cell death (Figure 7B and Figure S10A). In contrast, marginal zone (MZ.B) B cells, which typically express lower levels of IgD, exhibited more resistance to GR agonist treatment (Figure 7A,B). Although total cell numbers decreased across all B cell subpopulations, the reduction in follicular B cells was much more pronounced than that in marginal zone B cells, further highlighting the greater sensitivity of IgD^hi^ B cells to GR agonist treatment (Figure 7C,D).

As we previously observed that GR agonist treatment enhances activation and promotes plasma cell differentiation *in vitro*, we sought to determine whether the ratio of plasma cells was similarly increased *in vivo* in mice implanted with GC release pellets. Although the elevated expression of activation markers CD69 and CD86 was not detectable anymore after 14 days of treatment with GR agonists (Figure S10B), the ratio of plasma cells, identified by CD138 expression, was elevated in proportion to the potency of the GR agonist treatment (Figure 7E).

Additionally, IgM serum concentrations were significantly increased in correlation with the strength of the GR agonist effect (Figure 7F). These findings are consistent with the *in vitro* results, suggesting that IgD‐expressing B cells are particularly sensitive to GC‐induced cell death, whereas MZ‐like B cells, which may have regulatory functions, are relatively resistant. Together with the relatively reduced MZ population, despite the overall increase in total B cells in the conditional GR knockout (Figure 1A–C), our data suggest that GC control the differentiation and survival of B cells in BCR isotype‐dependent manner.

## 3 Discussion

Our study highlights the critical role of GR signaling in regulating B cell survival, differentiation, and immune function, confirming previous findings suggesting that the GR plays a role in regulating B cell homeostasis, possibly by eliminating certain B cell subsets that would otherwise persist in the absence of GR‐mediated signaling [38, 47, 48]. In full agreement, Ig‐λLC+ B cells are increased in the absence of GR signaling and this increase might be a result of unsuppressed NF‐κB activity as suggested previously [49].

Using both in vivo and in vitro models, we demonstrated that B cells with high IgD expression are particularly sensitive to GC‐induced cell death, whereas IgM^hi^ (IgD^lo^) B cells are relatively resistant, potentially acquiring a regulatory phenotype through increased IL‐10 expression [50, 51]. These findings provide important insights into how GCs modulate immune cell populations, thereby supporting and expanding existing literature on immunosuppressive effects elicited by GCs.

GR signaling has long been recognized for its potent immunosuppressive and anti‐inflammatory effects, with mechanisms that include apoptosis induction, regulation of cytokine production, and alteration of immune cell differentiation. Surprisingly, we observed that exposure to GCs promotes activation and plasma cell differentiation suggesting that B cells do not merely die by apoptosis but undergo terminal differentiation. This finding is in line with a previous study reporting increased expression of Blimp‐1, a key regulator of plasma cell differentiation, after the treatment of human B cells with GCs [40].

Early B cell developmental stages almost exclusively show IgM‐BCR expression, whereas later in development IgM‐ and IgD‐BCR are co‐expressed [52]. Although IgM‐ and IgD‐BCR share the proximal signaling machinery [53], both isotypes differ in structure, sensitivity to antigen valency [54] and interaction with co‐receptors due to differential organization in nanoclusters on the cellular membrane [55]. In our study, continuous GR agonist treatment selectively depleted IgD^hi^ follicular B cells, which are essential for germinal center responses [56], whereas sparing IgM^hi^ marginal zone‐like B cells.

This finding aligns with previous work, which described the ability of the GR to induce apoptosis in various immune cells, including T and B lymphocytes [38, 57, 58]. The selective depletion of IgD^hi^ B cells may reflect the differential sensitivity of these cells to GR‐induced death signals, which we observed both *in vivo* and *in vitro*.

Interestingly, although we did not detect significant differences in the expression of classical pro‐ or anti‐apoptotic genes between IgD^hi^ and IgM^hi^ B cells, we found that the latter exhibited an upregulation of genes such as *Il2rb* and *Cd28* (Table S11, positions 27 and 52, respectively). CD28 signaling has been shown to protect T cells from GC‐induced apoptosis, as described by Erlacher et al. [59], and our data suggest that a similar mechanism may play a role in B cells. The upregulation of CD28 in IgM^hi^ B cells may enhance their resistance to GC‐induced cell death, particularly when activated via TLR signaling pathways, as seen in our CpG and LPS stimulation experiments. This protective effect of CD28 and TLR activation echoes previous studies in T cells, where TCR and CD28 signaling antagonize GC‐mediated apoptosis [59, 60].

Our results further indicate that the activation state of B cells plays a crucial role in determining their sensitivity to GC‐induced apoptosis. Resting B cells were highly susceptible to GR‐mediated cell death, but TLR activation significantly improved survival. This is consistent with previous findings suggesting that TLR activation can protect plasmacytoid dendritic cells from GC‐induced apoptosis [61].

In B cells, TLR signaling not only enhances survival but also promotes the differentiation of IL10‐producing regulatory B cells (B10 cells), as reported previously [61]. These regulatory B cells, which we found to be enriched among IgM^hi^ populations, are crucial for controlling inflammation and maintaining immune homeostasis through their secretion of anti‐inflammatory cytokines like IL‐10 [50, 51].

The fact that treatment with GR agonists, in the presence of TLR stimulation, selects B cells with high IgM expression and increased IL‐10 production, highlights a potential mechanism through which GCs might selectively enrich regulatory cell populations. It is likely that this enrichment is due the sensitivity of IgD^hi^ (IgM^lo^) B cells to GC‐induced cell death. This finding is in agreement with published work, demonstrating that GCs promote the survival of anti‐inflammatory macrophages by activating specific signaling pathways [42]. In line with a previous report [40], the selective survival of IgM^hi^ B cells with increased IL‐10 expression found in our study suggests that a similar process may occur in B cells, where GR signaling promotes the enrichment of cells that might contribute to the resolution of inflammation.

Our study also suggests that the GR may exert its effects through both genomic and non‐genomic mechanisms. The lack of detectable changes in pro‐apoptotic genes like *Bim* or *Puma* in IgD^hi^ B cells despite their sensitivity to GR‐induced apoptosis suggests that GR may modulate cell death through non‐transcriptional pathways. This is supported by previous work showing that the GR can interact with signaling molecules, such as Lck and Fyn in T cells, disrupting key signal transduction pathways that are essential for cell survival [62]. Although our transcriptome analysis did not reveal direct involvement of these pathways in B cells, the upregulation of CD28 in IgM^hi^ cells hints at a broader role for GR in modulating B cell signaling networks.

Moreover, the role of TLR signaling in protecting B cells from GC‐induced apoptosis underscores the importance of innate immune signals in shaping the adaptive immune response during GC exposure. The differential kinetics of CpG and LPS activation, with CpG inducing faster and more robust activation, further supports the idea that the timing and strength of activation signals are critical for determining B cell fate under GC treatment. The delayed GR agonist treatment after LPS activation, which resulted in enhanced survival and plasma cell differentiation, highlights the potential for therapeutic strategies that modulate the timing of immune activation in conjunction with GC therapy.

In conclusion, our findings provide important insights into the selective effects of GR signaling on B cell subpopulations. By promoting the survival of IL‐10‐producing IgM^hi^ B cells and depleting IgD^hi^ follicular B cells, GR signaling may contribute to immune regulation and the resolution of inflammation. These results have broad implications for understanding the role of GCs in immune homeostasis and may inform the development of targeted therapies for autoimmune diseases and other inflammatory conditions.

## 4 Data Limitations and Perspectives

Although our study combines *ex vivo* assays with *in vivo* experiments to demonstrate elevated GC sensitivity in IgD‐expressing B cells, several limitations should be considered. First, our *in vivo* analyses relied on systemic GC administration and B cell‐specific GR deletion, which allowed us to investigate physiological endocrine exposure but may still underestimate the contribution of microenvironmental factors, that could differentially modulate GC responsiveness. Second, although our *in vivo* results support the enhanced sensitivity of IgD^+^ B cells, the temporal and dosage windows investigated were necessarily limited. We cannot exclude additional GC effects occurring at intermediate concentrations, at ultrashort exposure intervals, or during later phases. Third, the mechanistic basis underlying the differential sensitivity of IgD^+^ B cells remains only partially resolved. We focused on GR deletion and GC signaling readouts but did not comprehensively examine GR isoforms, co‐regulators, or post‐translational modifications. Similarly, long‐term consequences of GC exposure on B cell survival, repertoire stability, or function need to be addressed in further studies. Finally, exploring whether analogous mechanisms operate in human B cell subsets, particularly in patients undergoing GC therapy, represents a critical next step. Further addressing how endocrine signals shape B cell function remains an important next step increasing translational relevance and offering new opportunities for targeted immunomodulation.

## 5 Experimental Procedures

### 5.1 Mice

*GR^f/f^* mice (Nr3c1tm2Gsc/J, [63]) were crossed with *mb1‐cre* (CD79a‐cre) transgenic mice [64] to achieve B cell‐specific cre‐mediated deletion beginning at the early pro‐B cell stage. *GR^f/f^* × *mb1‐cre* and C57BL/6J WT mice were bred and housed the animal facility of Ulm University under specific‐pathogen‐free conditions. Mice aged 8–30 weeks were group‐housed with genetically distinct littermates of the same sex from weaning until experimental use. Both male and female mice were used in the study, except for GC pellet implantation studies. Absolute cell counts were determined using a LUNA FX7 automated cell counter.

GC‐releasing pellets (Innovative Research of America) containing Dex (3.5 mg/pellet) or Pred (0.35 mg/pellet) were implanted subcutaneously into male mice aged 11–22 weeks. All animal experiments were conducted in accordance with German animal welfare legislation and under license TVA1315 issued by the Regional Council Tübingen. Experimental procedures were approved by the Animal Care and Use Committees of Ulm University and the local authorities.

### 5.2 Cell Culture

Mature B cells were purified from murine spleens by MACS‐based positive or negative selection (CD19 MicroBeads, mouse; B cell isolation kit, mouse; both from Miltenyi Biotec) according to the manufacturer's instructions. Purified B cells were cultured in Iscove's modified Dulbecco's medium (Sigma‐Aldrich) supplemented with 10% heat‐inactivated FCS (PAN‐Biotech), 2 mM l‐glutamine (Gibco), 50 U/mL penicillin (Gibco), 50 U/mL streptomycin (Gibco), and 50 µM β‐mercaptoethanol (Gibco) at a density of 1–2.5 × 10^6^ cells/mL. Cells were stimulated either with 2.5 µg/mL LPS (Lipopolysaccharides from *Escherichia coli* O111:B4, Sigma‐Aldrich) or 2 µM CpG‐ODN #1826 (biomers) and treated with 25 nM Dex or Prednisolone (Selleckchem), respectively.

### 5.3 Flow Cytometry

Cell suspensions were blocked with α‐CD16/CD32 Fc‐Block and stained according to standard procedures with antibodies listed in Table S1. Viable cells were discriminated from dead cells by counterstaining with Fixable Viability Dye eFluor 450 or 780 (eBioscience, Thermo Fisher Scientific). For intracellular IL‐10 detection, cells were incubated with GolgiStop (1 µL/mL; BD Biosciences) for 4 h prior to fixation. Intracellular staining was performed using FIX&PERM Cell Fixation and Permeabilization Kit (ADG Nordic MUbio).

Biotinylated antibodies were detected with streptavidin (SAV)‐Qdot605 (Molecular Probes—invitrogen) or SAV‐eFluor 506 (eBioscience, Thermo Fisher Scientific). Data were acquired on a FACS Canto II, a FACS Lyric flow cytometer (BD Biosciences) or on a CytoFLEX S (Beckman Coulter) for the determination of absolute cell counts. Flow cytometry data were analyzed using FlowJo software version 10 (TreeStar). Unless otherwise indicated, numbers in dot plots represent the percentage of cells within the respective gates, whereas numbers in histogram plots correspond to the mean fluorescence intensity (MFI).

All flow cytometry experiments were performed in accordance with the *Guidelines for the use of flow cytometry and cell sorting in immunological studies* [65].

### 5.4 Enzyme‐Linked Immunosorbent Assay

96‐well MaxiSorp plates (NUNC) were coated either with 10 µg/mL polyclonal α‐mouse IgM or IgG antibody (SouthernBiotech) and subsequently blocked with PBS (Gibco) containing 1% BSA (Serva). Defined dilutions of mouse IgM or IgG antibodies (Southern Biotech) were used as standards. Serum samples were pre‐diluted 1:200 in blocking buffer and applied to the plate in duplicates using 1:3 serial dilutions.

IgM and IgG concentrations in serum were quantified using alkaline phosphatase‐labeled α‐mouse IgM or IgG detection antibodies (Southern Biotech). *P*‐nitrophenyl phosphate (Genaxxon) in diethanolamine‐buffer was added as substrate and absorbance was measured at 405 nm using a Multiskan FC plate reader (Thermo Fisher Scientific).

### 5.5 Cytometric Bead Array (CBA)

Mature splenic B cells from WT mice were purified and cultured *in vitro* for 4 days in the presence or absence of LPS. Supernatants from untreated cells were analyzed either undiluted or at a 1:2 dilution, whereas supernatants from LPS‐treated cells were pre‐diluted 1:2 or 1:10 and subjected to CBA analysis (Mouse IL‐10 Flex Set, BD Biosciences) according to the manufacturer's instructions.

### 5.6 RNA Sequencing

Splenic B cells were purified and cultivated as described in the cell culture section. After 3 days of treatment with LPS, B cells were FACS‐stained with α‐IgM and ‐IgD antibodies and sorted as shown in Figure S8A depending on their IgD expression into IgD^hi^ and IgM^hi^ B cell fractions by using a FACS Aria IIu cell sorter (BD Biosciences). Transcriptome analysis was performed by GENEWIZ Germany GmbH.

RNA sequencing (RNA‐seq) data were processed and analyzed using a series of steps starting with quality control and preprocessing. Raw paired‐end fastq files were trimmed for adapter sequences and low‐quality reads using Trimmomatic (version 0.39), with a sliding window approach and a minimum read length of 75 nucleotides. The cleaned reads were aligned to the *Mus musculus* (Mm) GRCm39 reference genome using Hisat2 (version 2.2.1), and the resulting alignments were converted to sorted BAM files using Samtools (version 1.9). Gene‐level counts were generated using FeatureCounts from Subread (version 2.0.6).

For differential gene expression analysis, the count data were imported into edgeR (version 3.32.1). To minimize the influence of lowly expressed genes, a filtering step was performed using the counts per million (CPM) values. Genes with CPM values greater than 8 in at least 6 samples were retained for further analysis. This threshold effectively removed genes with insufficient read counts across samples. Following this, the data were normalized using TMM (Trimmed Mean of *M* values) normalization to account for differences in library size.

Differential expression was assessed by fitting a generalized linear model (GLM) and performing likelihood ratio tests (LRT) on group comparisons. Genes with an absolute log‐fold change (log FC) ≥ 1.5 (corresponding to a fold change of ≥2) and an FDR < 0.05 were considered differentially expressed. To further refine the analysis, the glmTreat function was used to enforce the fold‐change threshold of 1.5.

KEGG pathway enrichment analysis was carried out in R using the kegga and topKEGG functions from the limma package. Entrez Gene identifiers from differentially expressed genes were used as input. Enrichment testing was performed with a species setting of Mm and an FDR threshold of 0.05.

### 5.7 Statistical Analysis

Graphs were created and statistical analysis was performed by using GraphPad Prism (Version 10) software. The number of individual replicates or mice (*n*) is stated in the figure legends or Tables S3–S10 as well as the tests applied to calculate statistical significance among observed differences. For analyses requiring paired testing (e.g., RM one‐way ANOVA or Friedman test), mice with missing data points at individual time points were excluded from the statistical analysis. *p* values < 0.05 were considered as statistically significant (ns = not significant; *p ≤ 0.05; ** *p* ≤ 0.01; *** *p* ≤ 0.001; ****p ≤ 0.0001).

## Author Contributions

Kais Almohammad, Mahmoud Alkhatib, and Corinna S. Setz performed mouse analyses, *in vitro* experiments, ELISA and flow cytometric measurements and data analyses. Kais Almohammad carried out ELISpot analyses, Marc Young analyzed deep sequencing data. Sabine Vettorazzi, Franziska Greulich, and Jan Tuckermann performed the *in vivo* treatment with GR agonist‐release pellets, provided the GR floxed mice and scientific input. Hassan Jumaa supervised the work and proposed the experiments. Kais Almohammad and Corinna S. Setz prepared the figures. Hassan Jumaa and Corinna S. Setz designed the study and wrote the manuscript. All co‐authors read and discussed the manuscript.

## Ethics Statement

All animal experiments were performed in accordance with institutional guidelines and approved by the responsible regional authority (Regierungspräsidium Tübingen, Germany; approval no. TVA1315).

## Conflicts of Interest

The authors declare no conflicts of interest.

## Supporting information




**Supporting File 1**: eji70137‐sup‐0001‐SuppMat.pdf.

**Supporting File 2**: eji70137‐sup‐0002‐tableS11.xlsx.

**Supporting File 3**: eji70137‐sup‐0003‐SuppMat.pdf.

## Data Availability

Data that support the findings of this study are available from the corresponding author upon reasonable request.
