# Supplementary material for: IgD‐Expressing Mature B Cells Exhibit Enhanced Sensitivity to Glucocorticoid‐Induced Cell Death
Source: Eur J Immunol. 2026 Jan 25;56(1):e70137. doi: 10.1002/eji.70137 (PMC12832068; doi:10.1002/eji.70137)
Supplement: Supplementary file 1 — Supporting File 1: eji70137‐sup‐0001‐SuppMat.pdf. [file EJI-56-e70137-s002.pdf]

Supplementary Figures

IgD-Expressing Mature B Cells Exhibit Enhanced Sensitivity to Glucocorticoid-Induced Cell Death

Kais Almohammad[1‡], Marc Young[1], Sabine Vettorazzi[2,3], Franziska Greulich[4], Mahmoud Alkhatib[1†], Jan Tuckermann[2,3], Hassan Jumaa[1*] and Corinna S. Setz[1*]

[1] Institute of Immunology; Ulm University Medical Center, 89081 Ulm, Germany
[2] Institute of Comparative Molecular Endocrinology, Ulm University, 89081 Ulm, Germany
[3] German Center for Child and Adolescent Health (DZKJ), partner site Ulm, 89081 Ulm, Germany.
[4] Chair of Metabolic Programming, TUM School of Life Sciences, ZIEL-Institute for Food & Health, Technische Universität München, 85354 Freising, Germany
[‡] Present address: Department of Cardiology, University Heart Center Ulm, 89081 Ulm, Germany
[†] Present address: Department of Neurology, Ulm University, 89081 Ulm, Germany

***Correspondence to**
Corinna Setz: phone: +49-731-500-65204, e-mail: corinna.setz@uni-ulm.de
Hassan Jumaa: phone: +49-731-500-65200, e-mail: hassan.jumaa@uni-ulm.de

Institute of Immunology, Ulm University Medical Center, D-89081 Ulm, Germany;
Fax: +49-731-500-65202

Supplementary experimental procedures

Flow cytometry.

Table S1 | Antibodies used in flow cytometric analyses

Antigen	Conjugate	Clone	Company
**CD1d**	APC	1B1	BioLegend
**CD5**	eFluor 450, PE or PE-Cy-7	53-7.3	eBioscience
**CD19**	PerCP-Cy5.5	1D3	BD Biosciences
**CD19**	Biotin	6D5	SouthernBiotech
**CD19**	eFluor 450	eBio1D3	eBioscience
**CD21/CD35**	APC or Biotin	7E9	BioLegend
**CD23**	PE	B3B4	BD Biosciences
**CD28**	PE	37.51	BioLegend
**CD43**	Biotin	S7	BD Biosciences
**CD45R (B220)**	PE-Cy7 or PerCP-eFluor 710	RA3-642	eBioscience
**CD69**	APC or Biotin	H1.2F3	eBioscience
**CD86**	PE-Cy7	GL1	BioLegend
**CD93**	APC	AA4.1	BioLegend
**CD122**	APC	5H4	BioLegend
**CD138**	PE	DL-101	eBioscience
**CD138**	Brilliant Violet 421	281-2	BioLegend
**GR**	Alexa Fluor 647	D8H2	Cell Signaling Technology
**GR Isotype ctrl**	Alexa Fluor 647	DA1E	Cell Signaling Technology
**IgD**	FITC, PE or Biotin	11-26	SouthernBiotech
**IgD**	APC	11-26	eBioscience
**IgM**	FITC	polyclonal	SouthernBiotech
**IgM**	eFluor 450	eB121-15-F9	eBioscience
**IgG_1**	Biotin	A85-1	BD Biosciences
**IgG_{2a}**	Biotin	R19-15	BD Biosciences
**IgG_{2b}**	Biotin	R12-3	BD Biosciences
**IgG_3**	Biotin	R40-82	BD Biosciences
**Ig-κLC**	Alexa Fluor 647	polyclonal	SouthernBiotech
**Ig-λLC**	PE	polyclonal	SouthernBiotech
**IL-10**	PE	JES5-16E3	BioLegend
**IL-10 Isotype ctrl**	PE	RTK4530	BioLegend

PCR.

Genomic DNA was isolated from purified spleen cell populations shown in **Fig. S1C** and PCRs for detection of the indicated alleles were performed by using the primers enlisted in **Table S2**. PCR products were separated on a 2% agarose gel and visualized by ethidium bromide staining.

Table S2 | Primer sequences

Gene	for (5' → 3')	rev (5' → 3')	Amplicon size [bp]
GR	cct tct cat tcc atg tca gca tgt	gtg tag cag cca gct tac agg a &<br>ggc atg cac att act ggc gtt ct	360 (deleted)<br>275 (floxed)<br>225 (wild-type)
mb1-cre	acc tct gat gaa gtc agg aag aac	gga gat gtc ctt cac tct gat tct	500
mb1-wt	ctg cgg gta gaa ggg ggt c	cct tgc gag gtc agg gag cc	400

ELISpot.

Murine splenic cells applied in the ELISpot assay were FACS-purified by using a FACS Aria IIu cell sorter (BD Bioscience), as shown previously (1). For detection of IgM 10 x 10^3, for IgG 50 - 100 x 10^6 cells were plated in triplicates. The assay was performed for IgM and IgG (ELISpot Basic ALP, Mabtech) according to manufacturer's instructions and pictures were acquired and analyzed by using an Astor ELISpot reader (Mabtech).

Cell culture.

Mature splenic B cells from WT mice were purified and cultured *in vitro* for 4 days in the presence or absence of 2.5 µg/mL LPS. Viability was assessed by culturing cells in the presence or the absence of recombinant murine 0.3 µg/mL IL-2 (biotechne, R&D Systems) or 10 ng/mL IL-10 (immunotools).

Statistics

Table S3 | Fig. 3B numbers of replicates

n =	percentages				absolute cell numbers			
	0 dpt	1 dpt	2 dpt	3 dpt	0 dpt	1 dpt	2 dpt	3 dpt
untreated	28	28	28	28	6	6	6	6
Dex		20	20	20		6	6	6
Pred		20	20	20		6	6	6

Table S4 | Fig. 3C numbers of replicates

n =	percentages					absolute cell numbers				
	0 dpt	1 dpt	2 dpt	3 dpt	4 dpt	0 dpt	1 dpt	2 dpt	3 dpt	4 dpt
LPS	23	23	23	22	23	6	6	6	5	6
LPS + Dex		23	23	22	23		6	6	5	6
LPS + Pred		23	23	22	23		6	6	5	6

Table S5 | Fig. 3D numbers of replicates

n =	percentages					absolute cell numbers				
	0 dpt	1 dpt	2 dpt	3 dpt	4 dpt	0 dpt	1 dpt	2 dpt	3 dpt	4 dpt
CpG	23	23	23	22	23	6	6	6	5	6
CpG + Dex		23	23	23	23		6	6	6	6
CpG + Pred		23	23	23	23		6	6	6	6

Table S6 | Fig. 4A numbers of replicates

CD69 n =	percentages					mean fluorescence intensity (MFI)				
	0 dpt	1 dpt	2 dpt	3 dpt	4 dpt	0 dpt	1 dpt	2 dpt	3 dpt	4 dpt
untreated	17	20	23	23	23	11	14	17	17	17
LPS		20	23	22	23		14	17	17	17
LPS + Dex		20	23	22	23		14	17	17	17
LPS + Pred		20	23	22	23		14	17	17	17

CD86 n =	percentages					MFI				
	0 dpt	1 dpt	2 dpt	3 dpt	4 dpt	0 dpt	1 dpt	2 dpt	3 dpt	4 dpt
untreated	17	23	23	23	23	11	17	17	17	17
LPS		23	23	22	23		17	17	17	17
LPS + Dex		23	23	22	23		17	17	17	17
LPS + Pred		23	23	22	23		17	17	17	17

Table S7 | Fig. 4B numbers of replicates

CD69 n =	percentages					MFI				
	0 dpt	1 dpt	2 dpt	3 dpt	4 dpt	0 dpt	1 dpt	2 dpt	3 dpt	4 dpt
untreated	17	20	23	23	23	11	14	17	17	17
CpG		20	23	22	23		14	17	17	17
CpG + Dex		20	23	23	23		14	17	17	17
CpG + Pred		20	23	23	23		14	17	17	17

CD86 n =	percentages					MFI				
	0 dpt	1 dpt	2 dpt	3 dpt	4 dpt	0 dpt	1 dpt	2 dpt	3 dpt	4 dpt
untreated	17	23	23	23	23	11	17	17	17	17
CpG		23	23	22	23		17	17	17	17
CpG + Dex		23	23	23	23		17	17	17	17
CpG + Pred		23	23	23	23		17	17	17	17

Table S8 | Fig. S5B numbers of replicates

CD69 n =	percentages			MFI		
	0 dpt	1 dpt	2 dpt	0 dpt	1 dpt	2 dpt
untreated	15	14	17	9	8	11
Dex		14	17		8	11
Pred		14	17		8	11

Table S9 | Fig. 5C numbers of replicates

n =	percentages			
	0 dpt	1 dpt	2 dpt	3 dpt
Dex (day 0)	28	20	20	20
LPS	27	23	28	28
LPS + Dex (day 0)		23	23	22
LPS + Dex (day 2)				21

Table S10 | Fig. 5F numbers of replicates

n =	percentages				
	0 dpt	1 dpt	2 dpt	3 dpt	4 dpt
-LPS control	17	17	21	21	21
+LPS control	17	17	21	20	21
+LPS +Dex				19	21

Supplementary Figures and Legends

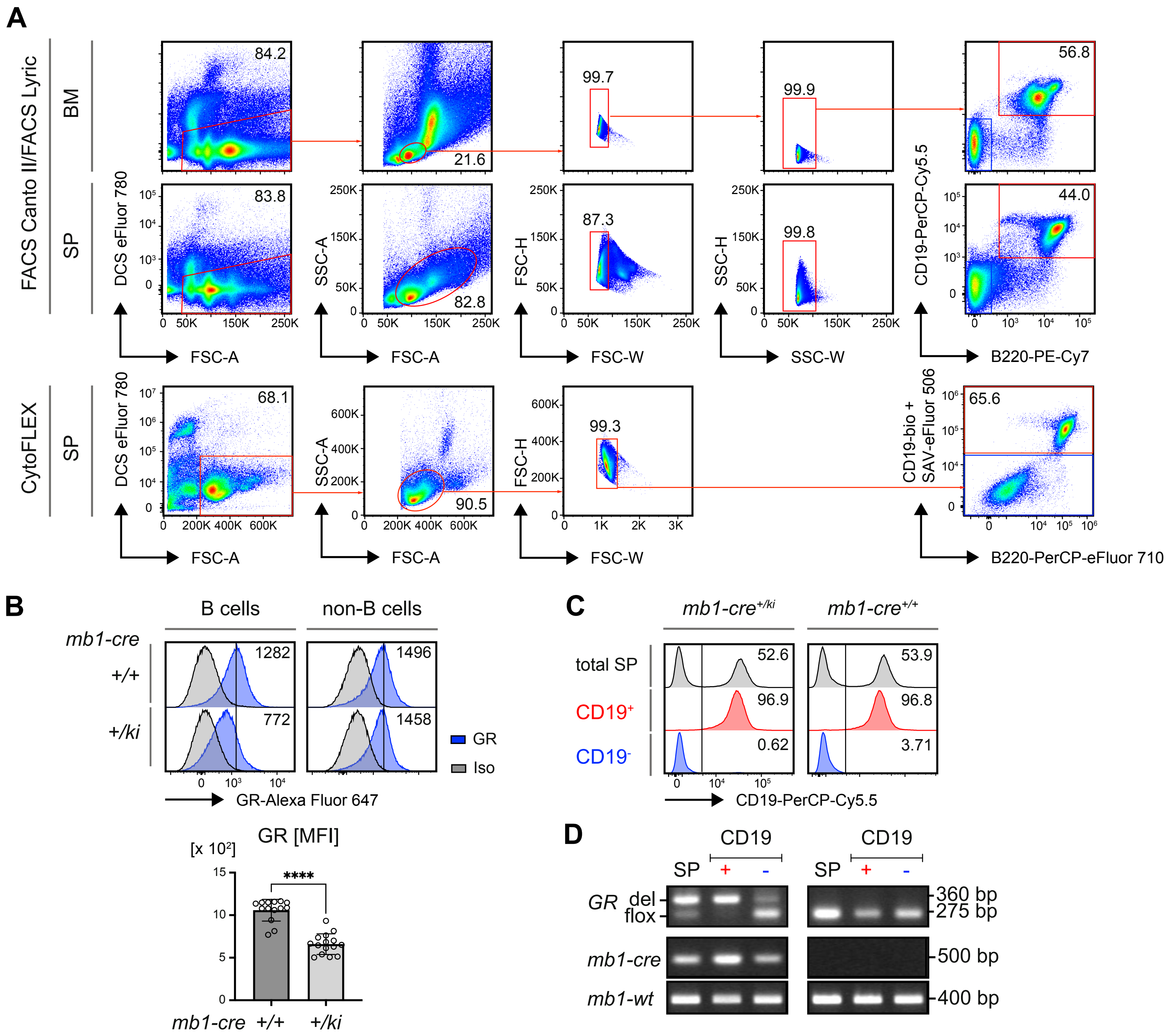

Figure S1 | Gating strategy and B-cell specific GR deletion

Figure S1 | Gating strategy and B-cell specific GR deletion

(related to main Figure 1)

A | General gating strategy applied for flow cytometric analysis of murine bone marrow (BM, top) and spleen (SP, middle) cells acquired on BD instruments (top and middle rows) and on CytoFLEX S (bottom row). DCS = dead cell stain.

B | Representative flow cytometric analysis (top) and quantified mean fluorescence intensity (MFI; bottom) of glucocorticoid receptor (GR) expression in splenic B- and non-B cell populations from 8-week-old *GR*$^{f/f}$ x *mb1-cre* mice of the indicated genotypes (*GR*$^{f/f}$ x *mb1-cre*$^{+/+}$: n = 14; *GR*$^{f/f}$ x *mb1-cre*$^{+/ki}$: n = 15). Data are presented as mean ± SD. Flow cytometric analyses were pre-gated on B cells as shown in (**A**). Statistical significance was determined using the unpaired Mann-Whitney-U test.

C | Purity analysis of spleen-derived CD19^{+} (red) and CD19^{-} (blue) populations, isolated via magnetic activated cell sorting (MACS)-based positive selection from *GR*$^{f/f}$ x *mb1-cre* mice of the indicated genotype. Numbers in histograms indicate percentages of positive cells. Flow cytometric analysis representative of three mice per genotype.

D | Genomic DNA from spleen cell populations shown in (**C**) was analyzed by PCR to detect the GR deleted (360 bp) and floxed (275 bp) allele, mb1-cre knock-in (ki, 500 bp), and mb1-wt (wt, 400 bp) allele. Data are representative of three mice per genotype.

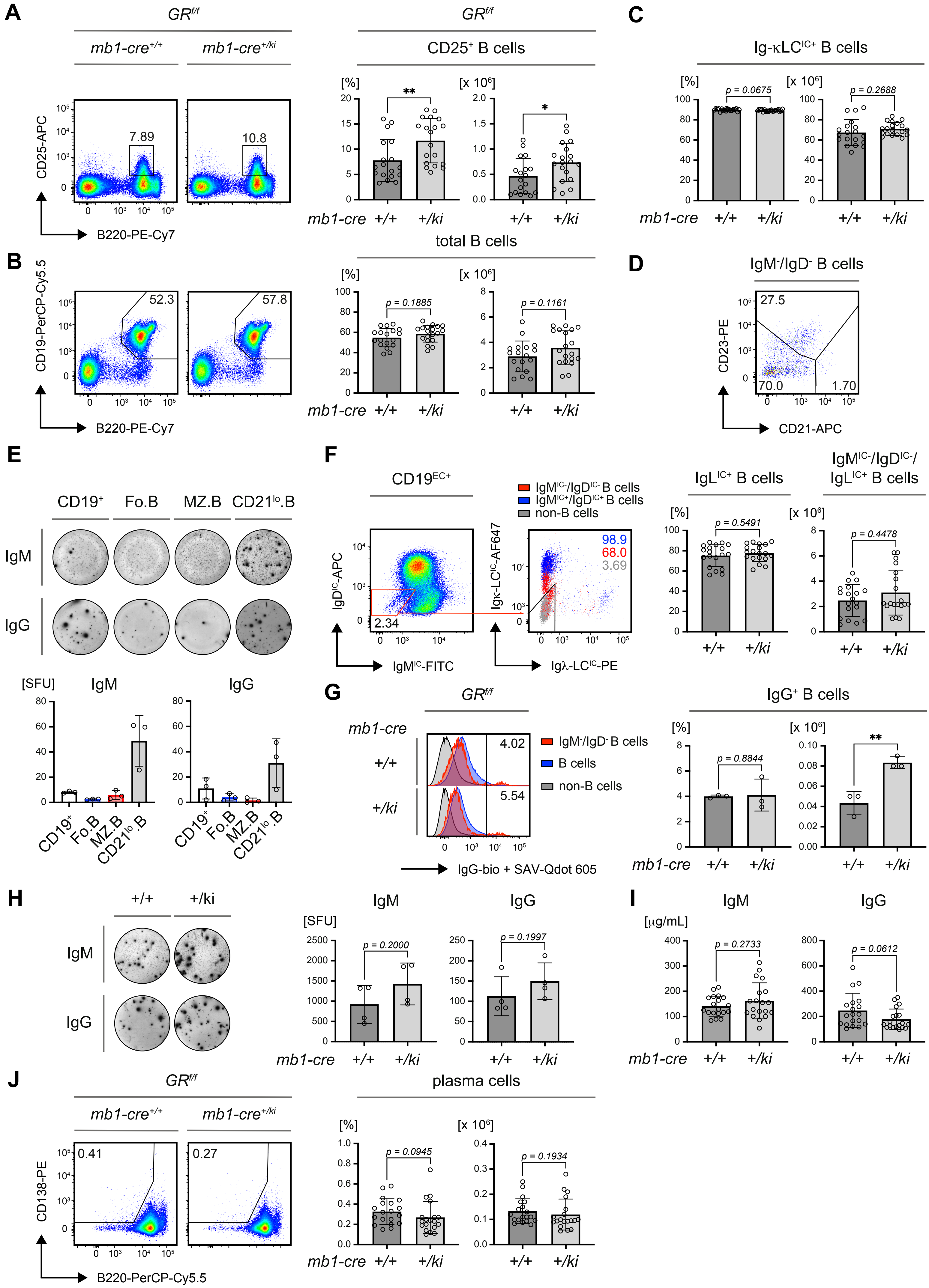

Figure S2 | B cell-specific GR deletion alters splenic B cell subpopulations

Figure S2 | B cell-specific GR deletion alters splenic B cell subpopulations

(related to main Figure 1)

Phenotype analyses of B cell populations in 8-week-old $GR^{f/f}$ x *mb1-cre* mice of the indicated genotype, n = 19 in each group, unless otherwise stated; mean ± SD. Flow cytometric data were pre-gated on B cells as shown in **Fig. S1A**. Unless specified otherwise, statistical significance was calculated by applying the unpaired t test.

A | Representative flow cytometric analysis (left panel) and quantification of both percentages (bar diagrams, left) and absolute cell numbers (bar diagrams, right) of small pre-B cells identified by CD25 expression in the BM from mice of the indicated genotypes. $GR^{f/f}$ x $mb1\text{-}cre^{+/+}$: n = 18; $GR^{f/f}$ x $mb1\text{-}cre^{+/ki}$: n = 19; mean ± SD. Statistical significance was calculated using the Mann-Whitney-U test for comparison of percentages.

B | Representative flow cytometric analysis (left panel) and quantification of both percentages (bar diagrams, left) and absolute cell numbers (bar diagrams, right) of total B cells identified by CD19 and B220 expression in the BM from mice of the indicated genotypes. $GR^{f/f}$ x $mb1\text{-}cre^{+/+}$: n = 18; $GR^{f/f}$ x $mb1\text{-}cre^{+/ki}$: n = 19; mean ± SD.

C | Quantified percentages of intracellular (IC) Ig-κLC^+ B cells (left panel) and absolute cell numbers (bar diagrams, right panel) in the spleens of mice from the indicated genotypes.

D | Representative flow cytometric analysis of CD21 and CD23 expression in IgM^-/IgD^- B cells derived from a $GR^{f/f}$ x $mb1\text{-}cre^{+/ki}$ mouse, pre-gated as shown in **Fig. 1F**.

E | Representative ELISpot data of IgM & IgG secreting cells in indicated B cell populations (left panel) purified from wild-type (WT) mice and quantification of spot forming units (SFU, right panel). n = 3; mean ± SD.

F | Representative flow cytometric analysis of IC Ig-κ & -λLC expression in IgM^-/IgD^- splenic B cells (red, left panel) and quantification of Ig-κ^+/-λLC^+ percentages within the IgM^-/IgD^- B cell population (bar diagrams, left) and absolute cell numbers of IgM^-/IgD^-/Ig-κ^+/-λLC^+ B cells (bar diagrams, right). Statistical significance was calculated by applying the Mann-Whitney-U test.

G | Representative flow cytometric analysis of IgG^+ B cells within the population of IgM^-/IgD^- splenic B cells (left panel) and quantification of percentages (bar diagrams, left) and absolute cell numbers (bar diagrams, right). Numbers in histograms indicate percentages of positive cells. n = 3; mean ± SD.

H | Representative ELISpot data of IgM & IgG secreting cells in FACS-purified splenic IgM^-/IgD^- B cell populations derived from $GR^{f/f}$ x *mb1-cre* mice of the indicated genotypes (left) and quantification of SFU (right), n = 4; mean ± SD.

I | Serum IgM (left) and IgG (right) concentrations in mice of the individual genotypes determined by ELISA. Statistical significance was determined by applying the Mann-Whitney-U test for comparison of IgG concentrations.

J | Representative flow cytometric analysis of plasma cells in the spleen (top panel), pre-gated on viable single $CD19^+$ cells and quantification of percentages (bar diagrams, bottom left) and absolute cell numbers (bar diagrams, bottom right). Statistical significance was calculated by applying the Mann-Whitney-U test.

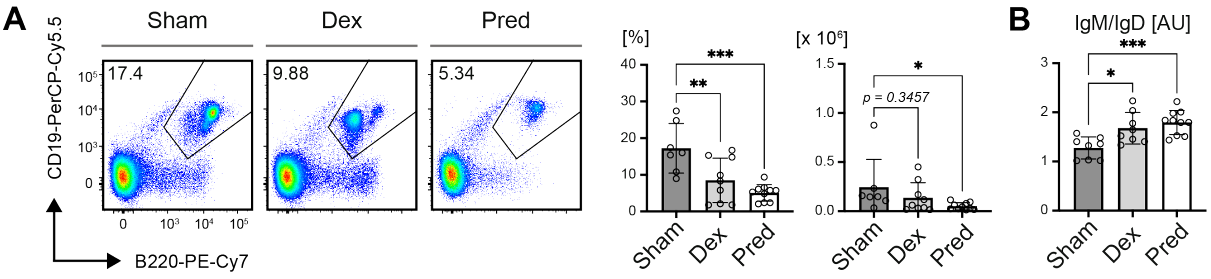

Figure S3 | Continuous GC-treatment eradicates B cells *in vivo*

(related to main Figure 2)

Phenotype analyses of mice transplanted with constant glucocorticoid (GC)-release pellets following 14 days of GC treatment.

A | Representative flow cytometric analysis (left panel) and quantification of both percentages (bar diagrams, left) and absolute cell numbers (bar diagrams, right) of total B cells in BM from mice after 14 days of exposure to Dexamethasone (Dex, n = 9), Prednisolone (Pred, n = 10) or control (Sham, n = 7) pellets. Mean ± SD. Statistical significance was calculated using the ordinary one-way ANOVA for percentages and the Kruskal-Wallis test for absolute cell numbers, respectively.

B | Quantified ratios of IgM/IgD surface expression (displayed as arbitrary units, AU) in splenic B cells from Sham- (n = 8), Dex- (n = 7) and Pred-treated (n = 9) mice. Values used for calculation are displayed in **Fig. 2F**. Mean ± SD. Statistical significance was calculated by using the ordinary one-way ANOVA.

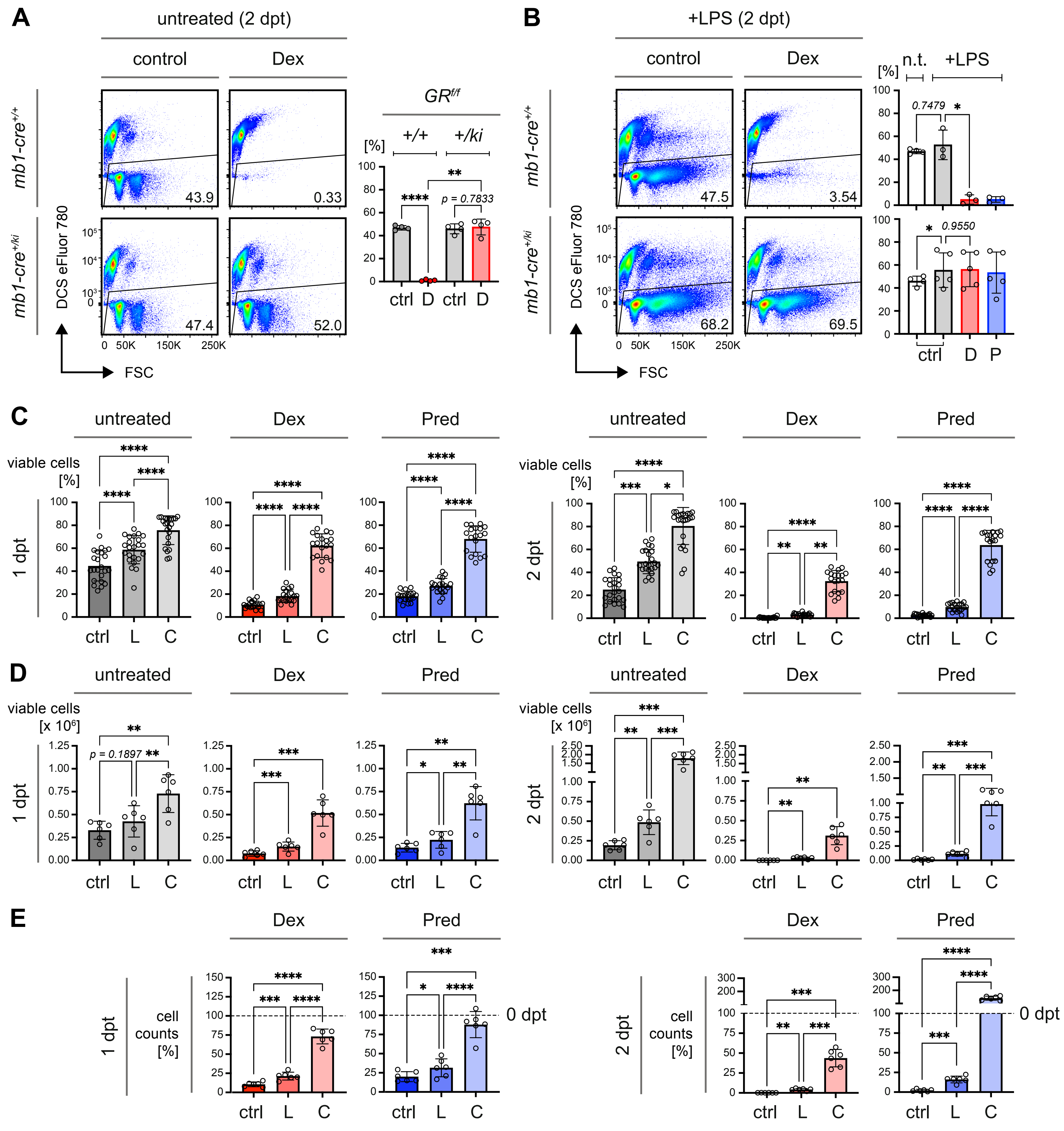

Figure S4 | Activated B cells display relative resistance to treatment with GR agonists

Figure S4 | Activated mature B cells are more resistant to GR agonist treatment *in vitro*

(related to main Figure 3)

A - B | Representative comparison (left) and quantification (right) of viability in splenic B cells from *GR*$^{f/f}$ x *mb1-cre* mice of the indicated genotypes in the presence (**B**, at least n = 3 for each group and genotype) or absence (**A**, n = 4 for each group and genotype) of lipopolysaccharide (LPS) upon treatment with GR agonists Dex (D) or Pred (P) at 2 days post treatment (dpt) *in vitro* as described in **Fig. 3A**; mean ± SD. Statistical significance was calculated by applying the repeated measures (RM) one-way ANOVA for **A** and the mixed-effects analysis for **B**.

C - D | Quantified percentages (**C**) and absolute cell numbers (**D**) of viable cells from **Fig. 3B-D** were compared at 1 (left panels) and 2 dpt (right panels) when treatment with GR agonists was performed in the presence of either LPS (L, **Fig. 3C**) or CpG (C, **Fig. 3D**) or without pre-stimulation (ctrl, **Fig. 3B**). Quantified percentages (right panel, top) and absolute cell numbers (right panel, bottom) of viable cells. Numbers of replicates are listed in **Table S3-5**, mean ± SD. Statistical significance was calculated by the repeated measures (RM) one-way ANOVA or Friedman test, respectively.

E | Relative change of absolute cell numbers at 1 dpt (left panel) and 2 dpt (right panel) normalized to day 0 values (indicated by dotted line), n = 6 per group, mean ± SD. Statistical significance was determined using the RM one-way ANOVA.

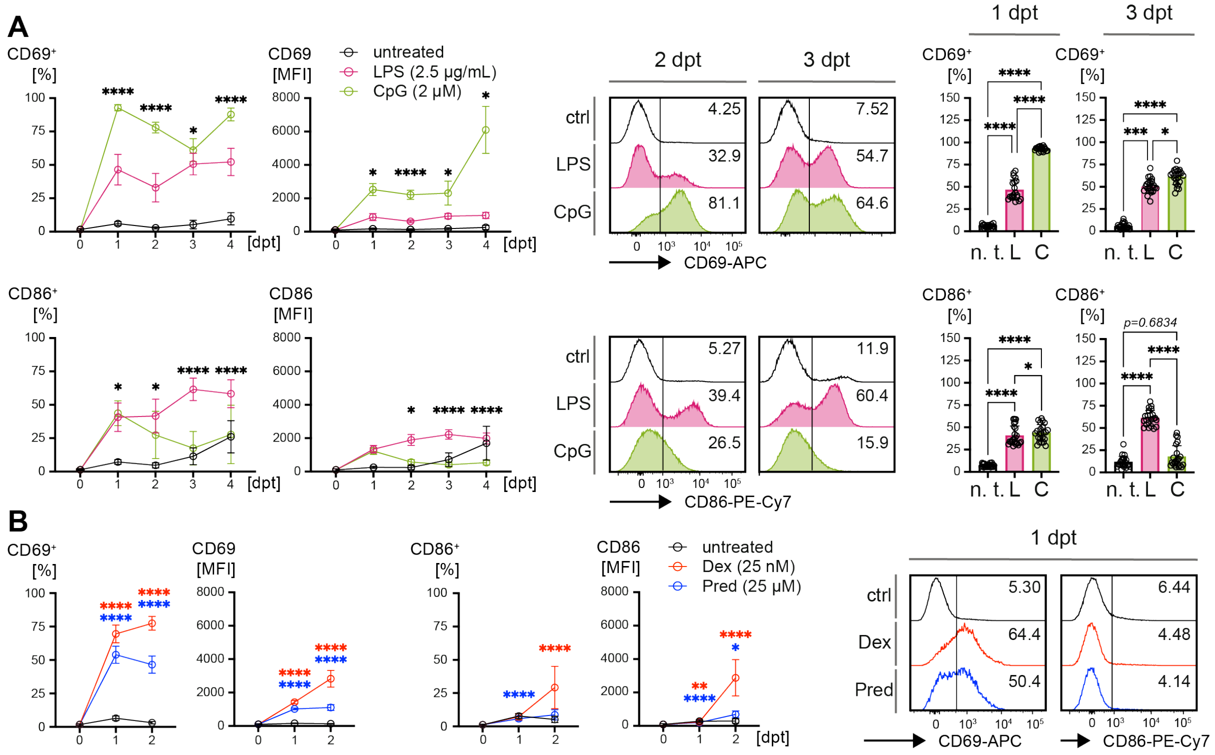

Figure S5 | GR agonists enhance B cell activation and accelerate terminal differentiation

(related to main Figure 4)

A | Kinetics comparing activation markers CD69 (top panel) and CD86 (bottom panel), determined by flow cytometry in WT splenic B cells following treatment with LPS and CpG, respectively. Representative flow cytometric analyses and bar diagrams on the right-hand side compare percentages and MFI of CD69 and CD86 positive cells upon LPS- or CpG-treatment at the indicated time points. Numbers in the histograms indicate percentages of positive cells. Data already shown as part of **Fig. 4A-B**. Numbers of replicates are listed in **Tables S6-7**; mean ± SD. Statistical significance was calculated by applying either the RM one-way ANOVA or the Friedman test, respectively. Asterisks in the kinetics indicate significant differences between cells stimulated either with LPS or CpG.

B | Kinetics of activation markers CD69 (left panels) and CD86 (middle panels), determined by flow cytometry in WT splenic B cells following treatment with the GR agonists Dex or Pred in the absence of LPS or CpG. Histograms on the right-hand side show representative flow cytometric analyses of CD69 and CD86 at 1 dpt. Numbers of replicates are listed in **Table S8**; mean ± SD. Numbers in histograms indicate percentages of positive cells. Statistical significance was calculated by applying either the RM one-way ANOVA or the Friedman test, as appropriate. Asterisks in the kinetics indicate significant differences between cells stimulated either with Dex or Pred and untreated controls.

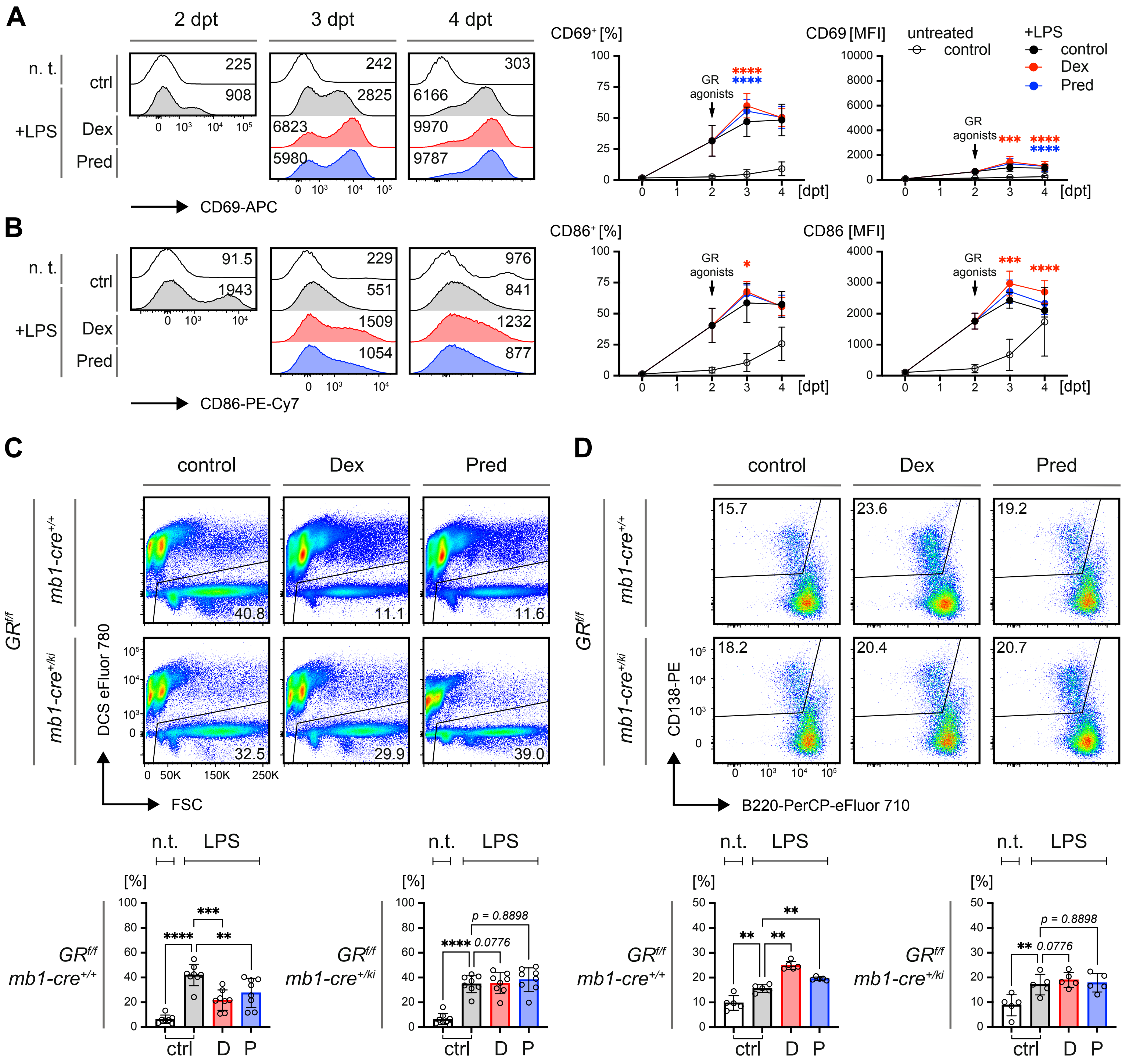

Figure S6 | Delayed GR agonist treatment after LPS activation improves B cell survival

Figure S6 | CD69^{hi} mature B cells are more robust to treatment with GR agonists *(related to main Figure 5)*

A - B | Representative flow cytometric analysis of activation markers CD69 (**A**) and CD86 (**B**) in WT splenic B cells at the indicated time points following treatment with GR agonists as described in **Fig. 5A** in the presence of LPS. Kinetics beside the histograms compare percentages of positive cells (left) and MFI (right) of CD69 (**A**) and CD86 (**B**). n = 11 - 21; mean ± SD. Statistical significance was calculated by applying the RM one-way ANOVA or the Friedman test. Mice with missing data points were excluded from the statistical analysis to allow for paired testing. Asterisks in the kinetics indicate significant differences between cells stimulated either with LPS and cells additionally treated with GR agonists Dex (D, red asterisks) or Pred (P, blue asterisks).

C | Representative flow cytometric analyses of cellular viability upon GC-treatment of purified splenic B cells derived from *GR*$^{f/f}$ x *mb1-cre* mice of the indicated genotypes at 3 dpt *in vitro* as described in **Fig. 5A**. Bar diagrams below show quantified of percentages of viable cells, respectively. Mean ± SD. Statistical significance was calculated for the *GR*$^{f/f}$ x *mb1-cre*$^{+/+}$ dataset (left, n = 7 for n. t. ctrl, n = 8 for all other groups) by applying the mixed-effects analysis with Šídák's multiple comparisons test and for the *GR*$^{f/f}$ x *mb1-cre*$^{+/ki}$ dataset (left, n = 9 for each group) by applying the RM one-way ANOVA, respectively.

D | Representative flow cytometric analyses of plasma cell differentiation in vitro upon GC-treatment of purified splenic B cells derived from *GR*$^{f/f}$ x *mb1-cre* mice of the indicated genotypes at 3 dpt as described in **Fig. 5A**. Bar diagrams below show quantified of percentages of CD19^{+}/CD138^{+} cells, respectively. n = 6 for all groups and genotypes, mean ± SD. Statistical significance was calculated by applying the RM one-way ANOVA, respectively.

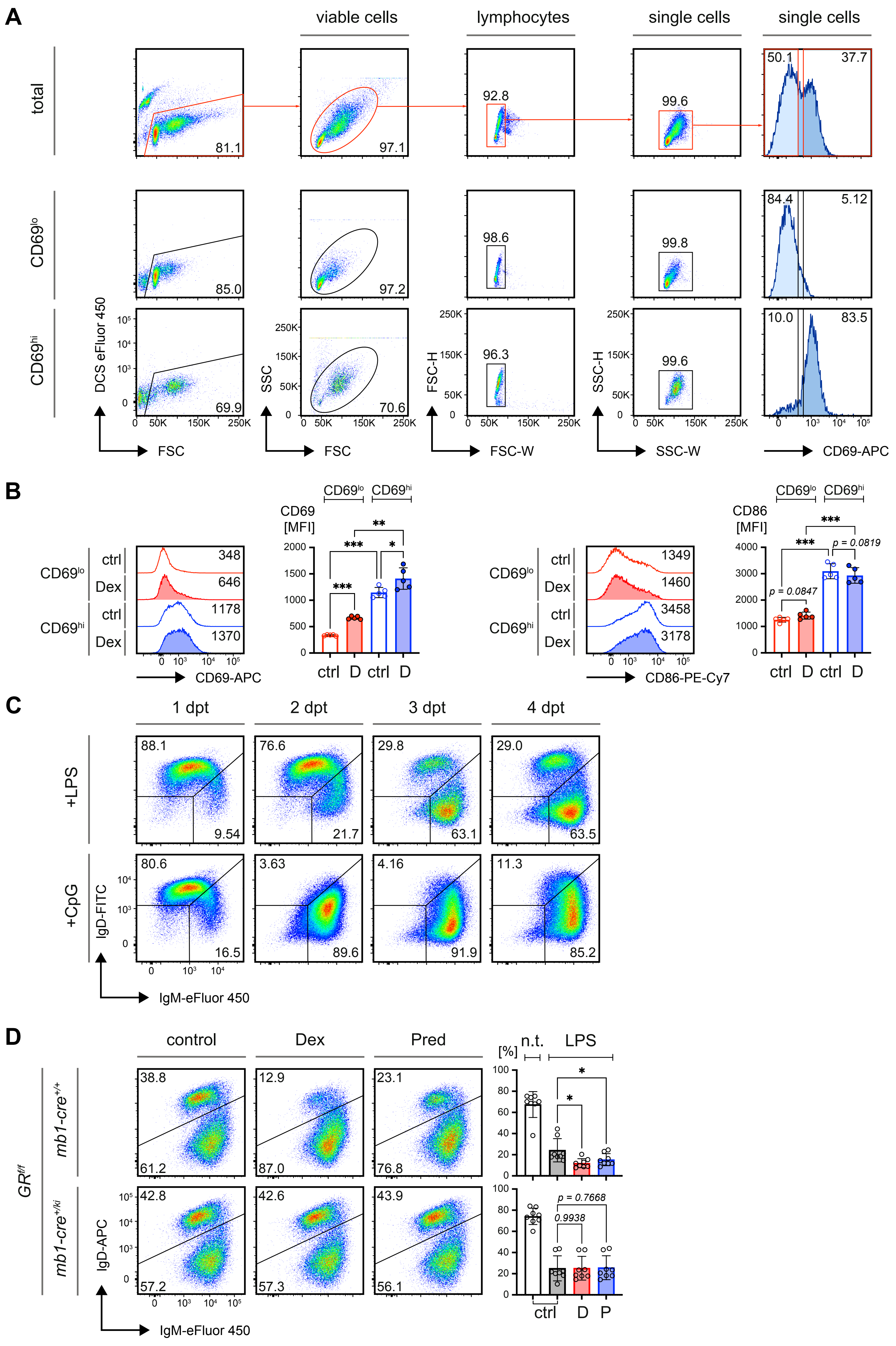

Figure S7 | IgD BCR expression affects resistance to cell death

Figure S7 | IgD BCR expression affects resistance to cell death

(A-B related to main Figure 5, C-D related to main Figure 6)

A | FACS-purification of $CD69^+$ and $CD69^-$ mature splenic B cells from WT mice. Mature splenic B cells were isolated via MACS-based negative selection and treated for 2 days with LPS. At 2 dpt, cells were stained for CD69 and FACS-purified according to the gating strategy, shown in the top row. Subsequently, the $CD69^{lo}$ and $CD69^{hi}$ populations were re-analyzed to assess purity. Representative data are shown in the two lower rows.

B | Representative flow cytometric analysis of activation markers CD69 and CD86 (left) and quantified MFI (right) in FACS-purified $CD69^{lo}$ and $CD69^{hi}$ mature splenic B cells from WT mice treated overnight in the presence or absence (ctrl) of Dex (D), n = 5, mean ± SD. Statistical significance was calculated by applying the RM one-way ANOVA.

C | Representative flow cytometric analyses of IgM and IgD BCR surface expression, determined at the indicated time points by flow cytometry in WT splenic B cells following treatment with LPS and CpG. Shown data are representative of at least 6 individual mice for all groups and time points, mean ± SD.

D | Representative flow cytometric analyses of IgM and IgD surface expression in purified splenic B cells derived from *GR*$^{f/f}$ x *mb1-cre* mice of the indicated genotypes at day 3 upon stimulation with LPS and delayed treatment with GCs as described in **Fig. 5A**. Bar diagrams show quantified percentages of IgM^{lo}/IgD^{hi} B cells cultivated in the presence of LPS and following GC treatment, n = 6 for all groups and genotypes, except for *GR*$^{f/f}$ x *mb1-cre*$^{+/ki}$, n. t. ctrl: n = 5. Statistical significance was calculated by applying the RM one-way ANOVA, respectively.

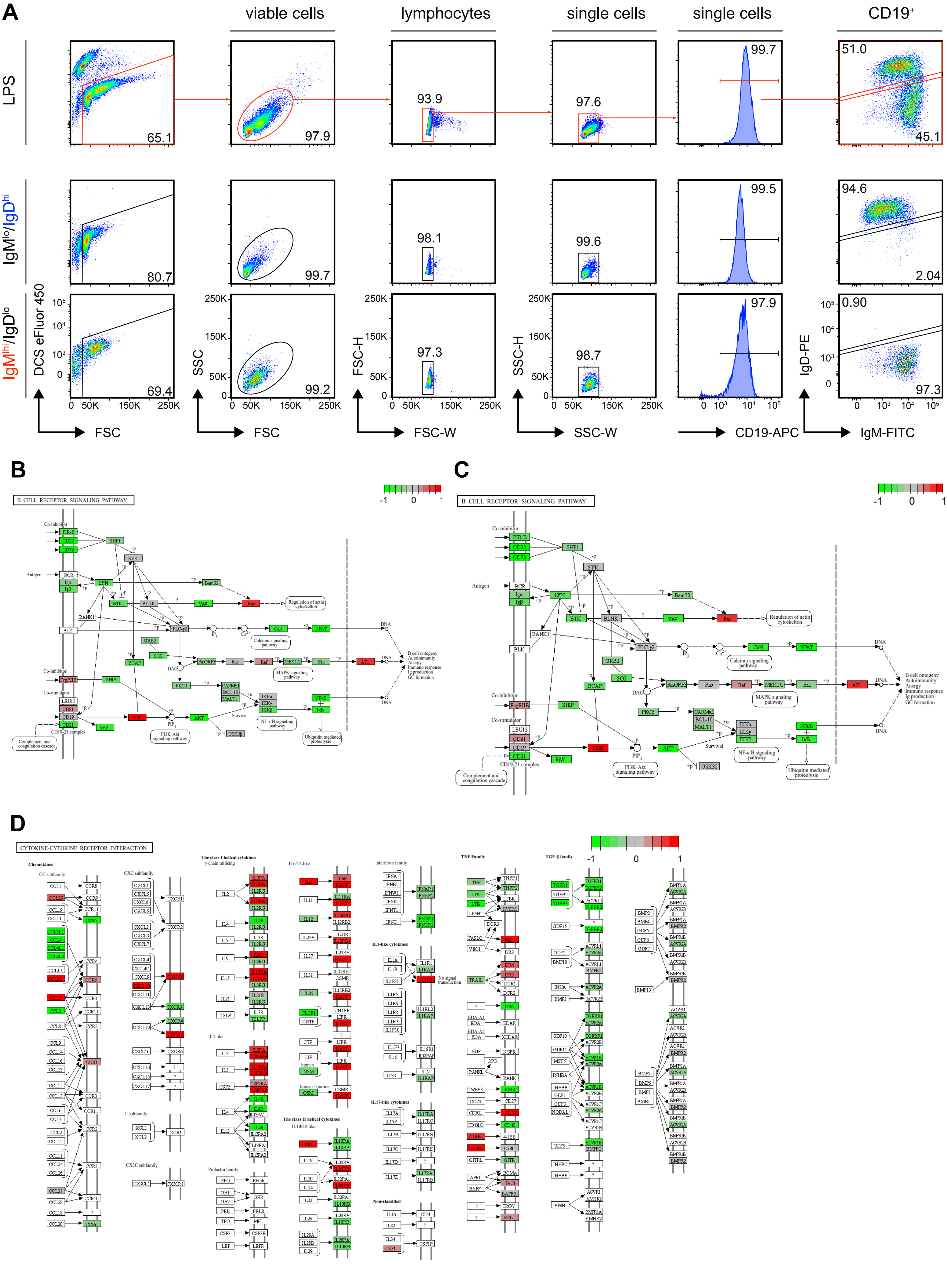

Figure S8 | Sorting strategies, gating and purity of IgD^{hi} and IgD^{lo} B cells

Figure S8 | Sorting strategies, gating and purity of IgD^{hi} and IgD^{lo} B cells *(related to main Figure 6)*

A | Gating strategy (top row) for FACS-purification of IgM^{lo}/IgD^{hi} and IgM^{hi}/IgD^{lo} B cell populations and representative purity (bottom rows) of sorted cell populations. B cells from WT mice were purified by MACS-based negative selection, and either stimulated with LPS or left untreated. After 2 days of incubation, cells were FACS-purified to separate IgM^{lo}/IgD^{hi} and IgM^{hi}/IgD^{lo} B cell populations.

B | KEGG-pathway analysis of B cell receptor (BCR) signaling in IgM^{lo}/IgD^{hi} and IgM^{hi}/IgD^{lo} B cell populations.

C | KEGG-pathway analysis of phosphoinositide 3-kinase (PI3K)-protein kinase B (PKB/AKT) signaling pathway in IgM^{lo}/IgD^{hi} and IgM^{hi}/IgD^{lo} B cell populations.

D | KEGG-pathway analysis of cytokine-cytokine receptor interactions in IgM^{lo}/IgD^{hi} and IgM^{hi}/IgD^{lo} B cell populations.

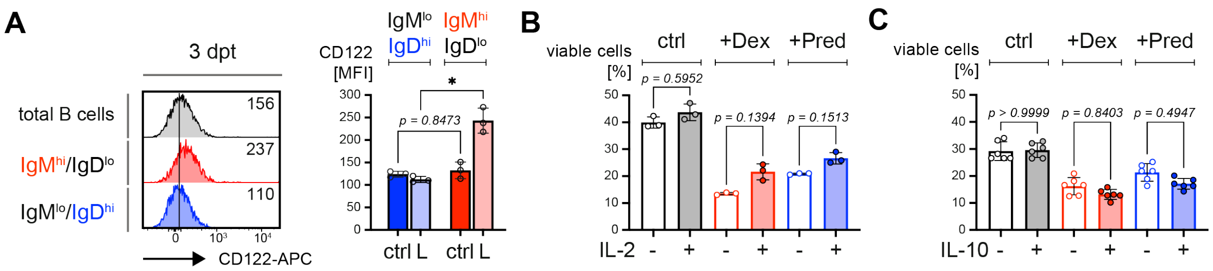

Figure S9 | IgD BCR expression affects resistance to cell death

(related to main Figure 6)

A | Representative flow cytometric analysis of CD122 expression in purified total mature B cells and IgM^{lo}/IgD^{hi} and IgM^{hi}/IgD^{lo} subpopulations upon treatment with LPS for 3 days (left). Quantification of CD122 MFI in IgM^{lo}/IgD^{hi} and IgM^{hi}/IgD^{lo} B cells. n = 3, mean ± SD. Statistical significance was calculated by applying the RM one-way ANOVA.

B | Quantification of mature B-cell survival in the presence or absence of IL-2 during exposure to GR agonists. Mature splenic B cells from WT mice were purified by MACS-based negative selection and treated with 2.5 µg/mL LPS. At 2 dpt, cells were treated with Dex or Pred in the presence or absence of 0.3 µg/mL IL-2. Percentages of viable cells were assessed by flow cytometry at 4 dpt. n = 3; data presented as mean ± SD. Statistical significance was calculated by applying the RM one-way ANOVA.

C | Quantification of mature B-cell survival in the presence or absence of IL-10 during exposure to GR agonists. Mature splenic B cells from WT mice were purified by MACS-based negative selection and treated with 2.5 µg/mL LPS. Cells were cultured with or without 10 ng/mL IL-10 throughout the experiment, and Dex or Pred were added at 2 dpt as described in (**B**). Percentages of viable cells were assessed by flow cytometry at 4 dpt. n = 6; data presented as mean ± SD. Statistical significance was calculated by applying the Friedman test.

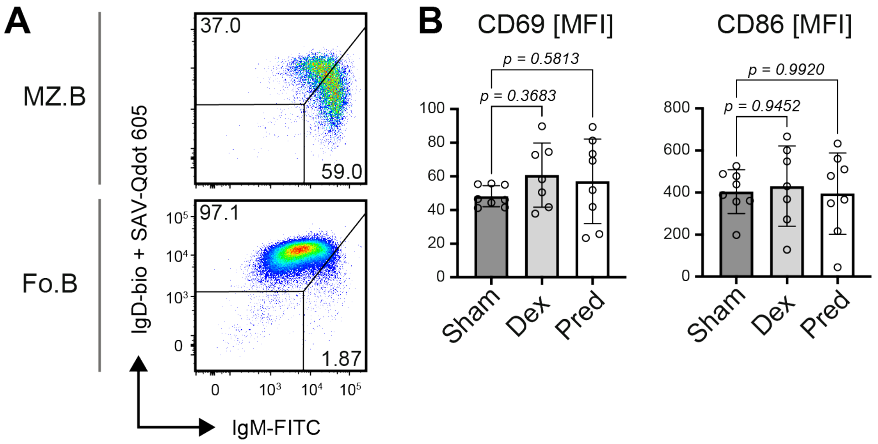

Figure S10 | Selective effects of GR agonist treatment on IgD expressing B cells *in vivo*

(related to main Figure 7)

Phenotype analyses of mice transplanted with constant glucocorticoid (GC)-release pellets after 14 days of GC treatment.

A | Representative flow cytometric analysis of IgM and IgD surface expression by follicular (Fo.B) and marginal zone B (MZ.B) cells purified from an untreated WT mouse.

B | Quantified MFI of CD69 (left) and CD86 MFI on splenic B cells from Sham- (n = 8), Dex- (n = 7) and Pred-treated (n = 8) mice. Mean ± SD. Statistical significance was calculated by using the ordinary one-way ANOVA.

Supplementary References

1. Setz CS, Hug E, Khadour A, Abdelrasoul H, Bilal M, Hobeika E, et al. PI3K-Mediated Blimp-1 Activation Controls B Cell Selection and Homeostasis. Cell Rep. 2018;24(2):391-405.
