# Supplementary material for: IgD‐Expressing Mature B Cells Exhibit Enhanced Sensitivity to Glucocorticoid‐Induced Cell Death
Source: Eur J Immunol. 2026 Jan 25;56(1):e70137. doi: 10.1002/eji.70137 (PMC12832068; doi:10.1002/eji.70137)
Supplement: Supplementary file 3 — Supporting File 3: eji70137‐sup‐0003‐SuppMat.pdf. [file EJI-56-e70137-s003.pdf]

ulm university universität ulm
Tierforschungszentrum
Prof. Dr. Kurt Reifenberg

Tel.: 0731-50-25581
Fax: 0731-50-25589

Universität Ulm | Tierforschungszentrum | 89081 Ulm | Germany

Brief Health monitoring report (based on FELASA recommendations 2014)

Unit: Bldg.N26, Room 1113 - 1118
Species: mouse
Housed strains/stocks: various strains and stocks
Housing type: Barrier/ IVC: rooms 1113 and 1118
Barrier/ open cages: rooms 1114, 1115, 1116, 1117

Date of report issue: 09.09.2025
Strain: colony animals from various strains

	Test frequency	Date of last results	Last results**	Testing laboratory	Test method	Historical results (≤ 18 months)
**Viruses**						
Mouse hepatitis virus (MHV)	3 months	Jul-25	0/13	TFZ	IFA	0/245
Mouse rotavirus (EDIM)	3 months	Jul-25	0/13	TFZ	IFA	0/245
Murine Norovirus (MNV)	3 months	Jul-25	0/13	TFZ	IFA	0/245
Parvoviruses:						
Minute virus of mice (MVM)	3 months	Jul-25	0/13	TFZ	IFA	0/245
Mouse parvovirus (MPV 1+2, VP2)	3 months	Jul-25	0/13	BioDoc	IFA	0/245
Theiler`s murine encephalomyelitis virus (TMEV)	3 months	Jul-25	0/13	TFZ	IFA	0/245
Lymphocytic choriomeningitis virus (LCMV)	annually	Jul-25	0/2	TFZ	IFA	0/143
Mouse adenovirus type 1+2 (MAD FL+K87)	annually	Jul-25	0/2	BioDoc	IFA	0/143
Ectromelia virus	annually	Jul-25	0/2	TFZ	IFA	0/143
Pneumonia virus of mice (PVM)	annually	Jul-25	0/2	TFZ	IFA	0/143
Reovirus type 3 (Reo3)	annually	Jul-25	0/2	TFZ	IFA	0/143
Sendai virus	annually	Jul-25	0/2	TFZ	IFA	0/143

ulm university universität ulm
Tierforschungszentrum
Prof. Dr. Kurt Reifenberg

Tel.: 0731-50-25581
Fax: 0731-50-25589

Universität Ulm | Tierforschungszentrum | 89081 Ulm | Germany

Brief Health monitoring report (based on FELASA recommendations 2014)

Unit: Bldg.N26, Room 1113 - 1118
Species: mouse
Housed strains/stocks: various strains and stocks
Housing type: Barrier/ IVC: rooms 1113 and 1118
Barrier/ open cages: rooms 1114, 1115, 1116, 1117

Date of report issue: 09.09.2025
Strain: colony animals from various strains

	Test frequency	Date of last results	Last results**	Testing laboratory	Test method	Historical results (≤ 18 months)
**Bacteria, Mycoplasma and Fungi**						
Helicobacter spp.	3 months	Jul-25	0/16	TFZ	PCR	0/312
Pasteurella spp.	3 months	Jul-25	0/16	TFZ	CULT / PCR	0/312
Rodentibacter heylii	3 months	Jul-25	0/16	TFZ	CULT / PCR	0/312
Rodentibacter pneumotropicus	3 months	Jul-25	0/16	TFZ	CULT / PCR	0/312
Strepococci ß-haemolytic (not group D)	3 months	Jul-25	0/16	TFZ	CULT	0/312
Streptococcus pneumoniae	3 months	Jul-25	0/16	TFZ	CULT	0/312
Citrobacter rodentium	3 months	Jul-25	0/16	TFZ	CULT / PCR	0/312
Clostridium piliforme	annually	Jul-25	0/2	BioDoc	ELISA	0/143
Corynebacterium kutscheri	3 months	Jul-25	0/16	TFZ	CULT	0/312
Mycoplasma pulmonis	annually	Jul-25	0/2	TFZ	IFA	0/143
Salmonella spp.	3 months	Jul-25	0/16	TFZ	CULT	0/312
Streptobacillus moniliformis	3 months	Jul-25	0/16	TFZ	CULT	0/312
Additional agents:						
Corynebacterium spp. (other than C. kutscheri)	3 months	Jul-25	0/16	TFZ	CULT	**1/312** *Jun 2024
Klebsiella pneumoniae	3 months	Jul-25	0/16	TFZ	CULT	0/312
Klebsiella oxytoca	3 months	Jul-25	0/16	TFZ	CULT	0/312
Pneumocystis murina	3 months	Jul-25	0/16	TFZ	PCR	0/312
Pseudomonas aeruginosa	3 months	Jul-25	**1/16**	TFZ	CULT	**14/312**
Staphylococcus aureus	3 months	Jul-25	0/16	TFZ	CULT	0/312
Strepococci ß-haemolytic (group D)	3 months	Jul-25	0/16	TFZ	CULT	0/312

ulm university universität uulm

Tierforschungszentrum
Prof. Dr. Kurt Reifenberg

Tel.: 0731-50-25581
Fax: 0731-50-25589

Universität Ulm | Tierforschungszentrum | 89081 Ulm | Germany

Brief Health monitoring report (based on FELASA recommendations 2014)

Unit: Bldg.N26, Room 1113 - 1118
Species: mouse
Housed strains/stocks: various strains and stocks
Housing type: Barrier/ IVC: rooms 1113 and 1118
Barrier/ open cages: rooms 1114, 1115, 1116, 1117

Date of report issue: 09.09.2025
Strain: colony animals from various strains

	Test frequency	Date of last results	Last results**	Testing laboratory	Test method	Historical results (≤ 18 months)
**Parasites**						
Ectoparasites:						
Fleas	3 months	Jul-25	0/16	TFZ	MICR	negative
Lice	3 months	Jul-25	0/16	TFZ	MICR	negative
Mites	3 months	Jul-25	0/16	TFZ	MICR	negative
Endoparasites:						
Giardia spp.	3 months	Jul-25	0/16	TFZ	MICR	0/312
Spironucleus spp.	3 months	Jul-25	0/16	TFZ	MICR	0/312
Entamoeba muris	3 months	Jul-25	0/16	TFZ	MICR	0/312
Chilomastix bettencourti	3 months	Jul-25	0/16	TFZ	MICR	0/312
Trichomonas spp.	3 months	Jul-25	0/16	TFZ	MICR	0/312
other flagellates	3 months	Jul-25	0/16	TFZ	MICR	0/312
Aspiculuris tetraptera	3 months	Jul-25	0/16	TFZ	MICR	negative
Syphacia spp.	3 months	Jul-25	0/16	TFZ	MICR	negative
**Pathological lesions observed**						
routine animal check (asymptomatic)	3 months	Jul-25	0/16	TFZ	PATH / CULT	0/312
asymptomatic screening (Pseudomonas aeruginosa)		Jan 24 -Feb 24	**21/62**	TFZ	CULT	

Data expressed as number positive/number tested
Abbreviation used in this report:
*** : date of last detectable result**
Serological samples and Helicobacter PCR samples are taken from both routine animal check and necropsy animal check
Positive findings in other species in the same unit:
TFZ=Tierforschungszentrum, D-Ulm, BioDoc=Biomedical Diagnostics, D-Hannover, MFD= mfd Diagnostics, D-Wendelsheim
ELISA= enzyme linked immunosorbent assay, MICR=microscopy, IFA=immunofluorescence assay, CULT=culture
PATH=gross Pathology, PCR=polymerase chain reaction, HIST=histopathology, NT=not tested
negativ = common results based on random sampling of the whole stock at TFZ

This report is valid without signature and stamp.
